# Genome size, chromosome number determination, and analysis of the repetitive elements in *Cissus quadrangularis*

**DOI:** 10.7717/peerj.8201

**Published:** 2019-12-20

**Authors:** Duncan Kiragu Gichuki, Lu Ma, Zhenfei Zhu, Chang Du, Qingyun Li, Guangwan Hu, Zhixiang Zhong, Honglin Li, Qingfeng Wang, Haiping Xin

**Affiliations:** 1 CAS Key Laboratory of Plant Germplasm Enhancement and Specialty Agriculture, Wuhan Botanical Garden, The Innovative Academy of Seed Design, Chinese Academy of Sciences, Wuhan, China; 2 Center of Economic Botany, Core Botanical Gardens, Chinese Academy of Sciences, Wuhan, China; 3 University of Chinese Academy of Sciences, Beijing, Peoples Republic of China; 4 Shenzhen Tobeacon Technology Co. Ltd., Shenzhen, Peoples Republic of China

**Keywords:** *Cissus quadrangularis*, Genome size, Copia, Repeat elements, Chromosome counts, Gypsy, Flow cytometry, C value

## Abstract

*Cissus quadrangularis* (Vitaceae) is a perennial climber endemic to Africa and is characterized by succulent angular stems. The plant grows in arid and semi-arid regions of Africa especially in the African savanna. The stem of *C. quadrangularis* has a wide range of applications in both human and animal medicine, but there is limited cytogenetic information available for this species. In this study, the chromosome number, genome size, and genome composition for *C. quadrangularis* were determined. Flow cytometry results indicated that the genome size of *C. quadrangularis* is approximately 2C = 1.410 pg. Fluorescence microscopy combined with DAPI stain showed the chromosome numbers to be 2*n* = 48. It is likely that *C. quadrangularis* has a tetraploid genome after considering the basic chromosome numbers in *Cissus* genus (*n* = 10, 11, or 12). A combination of low-throughput genome sequencing and bioinformatics analysis allowed identification and quantification of repetitive elements that make up about 52% of the *C. quadrangularis* genome, which was dominated by LTR-retrotransposons. Two LTR superfamilies were identified as Copia and Gypsy, with 24% and 15% of the annotated clusters, respectively. The comparison of repeat elements for *C. quadrangularis*, *Vitis vinifera,* and four other selected members in the *Cissus* genus revealed a high diversity in the repetitive element components, which could suggest recent amplification events in the *Cissus* genus. Our data provides a platform for further studies on the phylogeny and karyotype evolution in this genus and in the family Vitaceae.

## Introduction

*Cissus* is the largest genus in the grape family Vitaceae with about 300 species (Wen, 2007). The species in this genus show pan-tropical intercontinental disjunction, occurring in Asia, the Americas, Australia, and Africa (Jackes, 1988; Lombardi, 2015; Wen, 2007; Rodrigues, Lombardi & Lovato, 2014; Latiff, 2001). The greatest concentration of *Cissus* species is found in Africa with approximately 135 species; this is considered the ancestral area for this genus (Liu et al., 2013; Adams et al., 2016). The majority of research focuses on phylogenetic relationships within the genus and the grape family (Liu et al., 2013; Rodrigues, Lombardi & Lovato, 2014; Wen et al., 2018). The tribe Cisseae contains only *Cissus*, which is based on the new phylogenetic tribal classification of Vitaceae (Wen et al., 2018).

*Cissus quadrangularis* is one of the perennial succulent plants within *Cissus* that is widely distributed in Africa, the Arabian Peninsula, northern India, and Southeast Asia (GBIF Backbone Taxonomy). Ting, Sternberg & Deniro (1983) identified a facultative crassulacean acid metabolism (CAM) pathway in *C. quadrangularis* that could contribute to its excellent tolerance to drought conditions. Scientific interest in this species has increased recently due to its value in veterinary and human medicine (Latiff, 2001; Mishra, Srivastava & Nagori, 2010; Stohs & Ray, 2012; Ganguly, Ganguly & Banerjee, 2018; Vedha, Bn & Devi, 2013; Indran & Raj, 2015).

Previously reported chromosome numbers for *Cissus quadrangularis* have been inconsistent. Raghavan (1957) examined the chromosome number for Indian medicinal plants and determined the chromosome numbers for *C. quadrangularis* to be 2*n* = 24. He suggested that earlier reports of a diploid with 45 chromosomes could have been incorrect or that the specimen was possibly a tetraploid. Further, Robert et al. (2001) examined *C. quadrangularis* from Kenya and its two variants (A & B) and determined their chromosome numbers to be 24 and 28, respectively. Variant A was identified with smooth stem angles and was proposed to be the type variety for *C. quadrangularis* while variant B had rough stem angles and was considered to be a new variety of *C. quadrangularis*. Karkamkar, Patil & Misra (2010) reported the chromosome numbers of seven *Cissus* species (2*n* = 24, 48). In addition, Chu et al. (2018) reported the chromosome numbers for seven other *Cissus* species (Table 1, 2*n* = 24, 40, 48, or 66) and suggested a linear relationship between the chromosome numbers and genome size (1C = 0.37–1.03 pg). These results implicate polyploidization and repetitive element modifications in the expanded genome size in this genus. However, considering the large number of species in the *Cissus* genus, much is unknown about the genome size, chromosome numbers, and genome characteristics of the members comprising this genus.

**Table 1:** Chromosome numbers, genome size and ploidy types in *Cissus*. *Cissus quadrangularis* data was obtained in the present study. Data for other members have been documented by Chu et al. (2018), Robert et al. (2001) & Raghavan (1957).

Cissus species	Chromosome numbers (2*n*)	Ploidy type	Genome size (2C-Value)
*C. rotundifolia* Vahl	24	2 ×	0.76 pg.
*C. discolor* Blume	24	2 ×	0.86 pg.
*C. tuberosa* Moc. & Sesse ex DC.	24	2 ×	0.9 pg.
*C. javana* DC.	–	–	0.74 pg.
*C. antarctica* Vent	40	4 ×	1.34 pg.
*C. trifoliata (L.) L*	48	4 ×	1.58 pg.
*C. microcarpa* Vahl	66	6 ×	2.06 pg.
*C. quadrangularis-*Raghavan (1957)	24	2 ×	–
*C. quadrangularis*-Robert et al. (2001)	24, 28	–	–
*C, quadrangularis-* This study	48	–	1.410 pg

Repetitive sequences form up to 90% of the plant genome and are dominated by long terminal repeats (LTR) in plants (Du et al., 2010). The disparity in the plant genome size is attributed to polyploidisation events and the variation in the amount of repetitive DNA, which is characterized by transposable elements and tandem repeats (Kidwell, 2002; Sessegolo, Burlet & Haudry, 2016). Mis-annotation of LTR has led to the characterization of some of LTR as genes, especially the low copy number fragments (Bennetzen et al., 2004). This makes the complete and accurate annotation of transposable elements in whole genome sequencing projects of plants necessary. Macas, Neumann & Navrátilová (2007) utilized short read sequencing technology to identify repeat elements without using a reference genome based on the similarity of their reads. Sequencing reads can be classified into clusters based on their similarities representing the repetitive elements.

The advent of robust sequencing technologies that can generate huge sequence data at a reduced cost, coupled with advanced assembling methods, has improved genome sequencing for both model and non-model plants (Michael & VanBuren, 2015). Whole genome sequencing projects are challenging due to a limited amount of data. Additionally, proportions of repetitive DNA components in the genome can impede sequencing. In maize, repetitive elements form about 80% (SanMiguel et al., 1996) of the genome with a complex organization that created difficulty when sequencing its whole genome (Chandler & Brendel, 2002). Challenges in incorporating repetitive DNA sequence data have been one of the limiting factors in the available draft genomes (Feuillet et al., 2011).

Kidwell (2002) reported that there is a closer relationship between repetitive DNA sequences and genome size. Li et al. (2017) confirmed a positive relationship between genome size and repetitive sequences. Their analysis revealed a stronger positive correlation between retrotransposons and genome size than with transposons. Among the retrotransposons, LTR-retrotransposons were shown to have the highest positive correlation. However, the contributions of LTR lineages (Ty1-Copia and Ty3-Gypsy) to the genome size were similar. Understanding the components of the repetitive elements will therefore undoubtedly provide clues about the factors that may have influenced the expanded genome in *C. quadrangularis* and may include multiplication of its repetitive elements.

In the present study, the chromosome numbers and genome size of *C. quadrangularis* were evaluated. Short-read sequencing data were generated from the genomic DNA of *C. quadrangularis* to characterize its major genomic components and the fractions of repetitive elements. Comparisons were made between the repetitive element components for *C. quadrangularis*, *Vitis vinifera*, and four other *Cissus* species (Table 1). The findings reported here increase our understanding of the genome variations in the *Cissus* genus and provide basic genomic and cytogenetic information for *C. quadrangularis* that forms a foundation for whole genome sequence studies.

## Materials and Methods

### Plant materials

Stem cuttings of *C. quadrangularis* were collected from the roadside in Namango, Kenya, (S02°32′, E36°49′). Duplicate voucher specimens (SAJIT 002306) were deposited in the Wuhan Botanical Garden herbarium (HIB) and the Herbarium of Jomo Kenyatta University of Agriculture and Technology (JKUAT). Young leaves and petioles were collected for genome size evaluation while root tips were collected for chromosome number determination. All materials for the genome sequencing and karyotyping of *C.quadrangularis* were obtained from a single individual.

### Genome size estimation

Flow cytometry was used to determine nuclear DNA content with minor modifications using hand chopped material as originally described by Galbraith et al. (1983). In order to isolate the nuclei, a woody plant buffer (WPB) was used, which contained 0.2 M Tris-HCl, 4 mM MgCl_2_⋅6H_2_O, 2 mM EDTA Na_2_⋅2H_2_O, 86 mM NaCl, 10 mM sodium metabisulfite, 1% PVP-10, 1% (v/v) Triton X-100, with a pH 7.5 (Loureiro et al., 2007a; Loureiro et al., 2007b). *Raphanus sativus* cv. Saxa (radish) seeds provided by the Institute of Experimental Botany, Czech Republic, were germinated and the plantlets were used as reference standards. The plantlets of *C. quadrangularis* were pre-treated for 4 days in the dark. Petioles from the young leaves of *C. quadrangularis* and young leaves from radishes were collected (approximately 50 mg for each sample) and hand chopped using a sharp razor on ice in 1.5 mL WPB as described by Pfosser et al. (1995). The suspension was filtered through a 40-µm-nylon mesh (Cat. 352340, Falcon, USA) to eliminate the excess debris. RNase A was added to 100 ng/mL in the nuclei homogenate solution. Propidium Iodide (50 mg/mL) was used to stain the nuclei for at least 2 min on ice before the samples were run in the flow cytometer (BD Accuri C6). Three independent samples were run on the cytometer and the genome size was calculated using the following formula: sample 2C DNA content = [(sample G1 peak mean)/(internal standard peak mean)] × Internal standard DNA content.

### Chromosome count

Root tips were obtained from *C. quadrangularis* plantlets propagated from the same individual and collected from the field. Samples were treated with a saturated solution of 1-Bromonaphthalene for 3 h at room temperature to halt cell division (Mirzaghaderi, 2010). Microscopic slides were then prepared from the treated root tips using the protocol as developed by Kirov et al. (2014) with minor modifications to obtain the chromosomes at metaphase stage. To digest the cell wall, the root tips were incubated for 60 min at 37 °C in a 1% enzyme mix (1% pectinase and 1% cellulase in freshly prepared 0.1 M citrate buffer). Relative humidity was maintained between 40 and 60% during the dropping step. The prepared microscopic slides were stained with 4′, 6-diamidino-2-phenylindole (DAPI) and chromosome fluorescent images were captured using a fluorescence microscope (Leica DMi8) fitted with a camera (Leica DFC 550).

### DNA isolation, sequencing, and data analysis

Genomic DNA was extracted from the leaves of *C. quadrangularis* using the plant genomic DNA kit (Tiangen, China) following the manufacturer’s protocol. DNA libraries were constructed the using NEBNext® Ultra™ DNA Library Prep Kit for Illumina. Paired-end sequences (2 × 150 bp and 400–450 bp insert size) were generated on the Illumina HiSeq X Ten platform produced by Novogene (Tianjing, China). The sequencing data were deposited in the SRA database under accession number SRR8573652.

To analyze the repetitive components of this species, graph-based clustering was performed using the RepeatExplorer platform (Novák, Neumann & Macas, 2010). Short-read sequencing data was subjected to the Galaxy-based RepeatExplorer platform as described by Novak et al. (2013). The quality of the reads was determined by the FastQC tool and poor-quality reads were discarded. Clean reads were converted into the FASTA format Using the FASTQ to FASTA converter. It was possible to infer the repeat composition for a species with typically 0.1–0.5× genome coverage using the RepeatExplorer (Macas et al., 2015). A total of 700,000 paired-end reads (150 bp) were randomly selected, which were equal to approximately 6.56% of the predicted genome for clustering. An all-to-all comparison was carried out, grouping similar sequences into respective clusters in RepeatExplorer. The genome proportions for each cluster were calculated based on the read percentages.

Repeat clusters contributing no less than 0.01% of the genome proportions were considered for further annotation while those with smaller contributions were ignored. Repeat clusters with known protein domains were annotated directly on the RepeatExplorer platform, while similarity searches against GenBank databases (nt and nr) using BLASTn and BLASTx (Altschul et al., 1990) were carried out manually with the *E*-value at 1e^−5^ to classify other repeat clusters.

### Comparison of repeat content in *C. quadrangularis*, grape, and 4 other *Cissus* species

To investigate variations in repeat elements among the species and their roles during the evolution of the *Cissus* genus, we collected short-read sequencing data for other *Cissus* species, which is publicly available at NCBI. Four *Cissus* species whose genome size and sequencing data are available (*C. tuberosa, C. trifoliata, C. discolor, and C. microcarpa*), were selected for co-clustering (last accessed May 20, 2019). Sequencing and genome size data are essential in determining the proper number of reads representing similar genome proportions. Co-clustering analysis was performed using RepeatExplorer (Novak et al., 2013). Reads from *C. quadrangularis*, four other *Cissus* species and grape were simultaneously clustered. The species were randomly grouped and considered to have equal probabilities for common repeats and frequencies among the 6 species. Co-clustering allowed the grouping of similar reads from individual species suggesting similar ancestral origin. In order to avoid a sensitivity bias, the number of reads that were analyzed for all species are proportional to the genome sizes of the corresponding species. Randomly selected paired-end reads from *C. quadrangularis*, grape, *Cissus microcarpa, Cissus trifoliata, Cissus discolor, and Cissus tuberosa*, (ca. 0.06× coverage for each genome) were combined for RepeatExplorer co-clustering (Novak et al., 2013). The sources for clustering data are provided in Table 2. The genome size data used for annotation of repeats for *Cissus* species has been reported by Chu et al. (2018). Plastid repeats are phylogenetically uninformative due to their abundance, which is linked to the photosynthetic dynamics in plant tissues (Dodsworth et al., 2016) and were manually excluded in further analysis.

**Table 2:** Genomic and co-clustering data for *Cissus quadrangularis*, Grape (Pinot noir) (*Vitis vinifera*) and four other *Cissus* species.

Species	Data source	Genome size (1C)	Length of reads	6% in reads number	Reads used
*Cissus quadrangularis*	This study	689	130	318000	318000
*Vitis vinifera*	SRR5627797	475	130	219230.7692	219000
*Cissus discolor*	SRX1323033	420.54	130	194096.3846	194000
*Cissus microcarpa*	SRX1322892	1007.34	130	464926.1538	465000
*Cissus trifoliata*	SRX1322890	772.62	130	356593.8462	357000
*Cissus tuberosa*	SRX1322889	440.1	130	203123.0769	203000

## Results and Discussion

### C-value determination in *Cissus quadrangularis*

**Figure 1:**
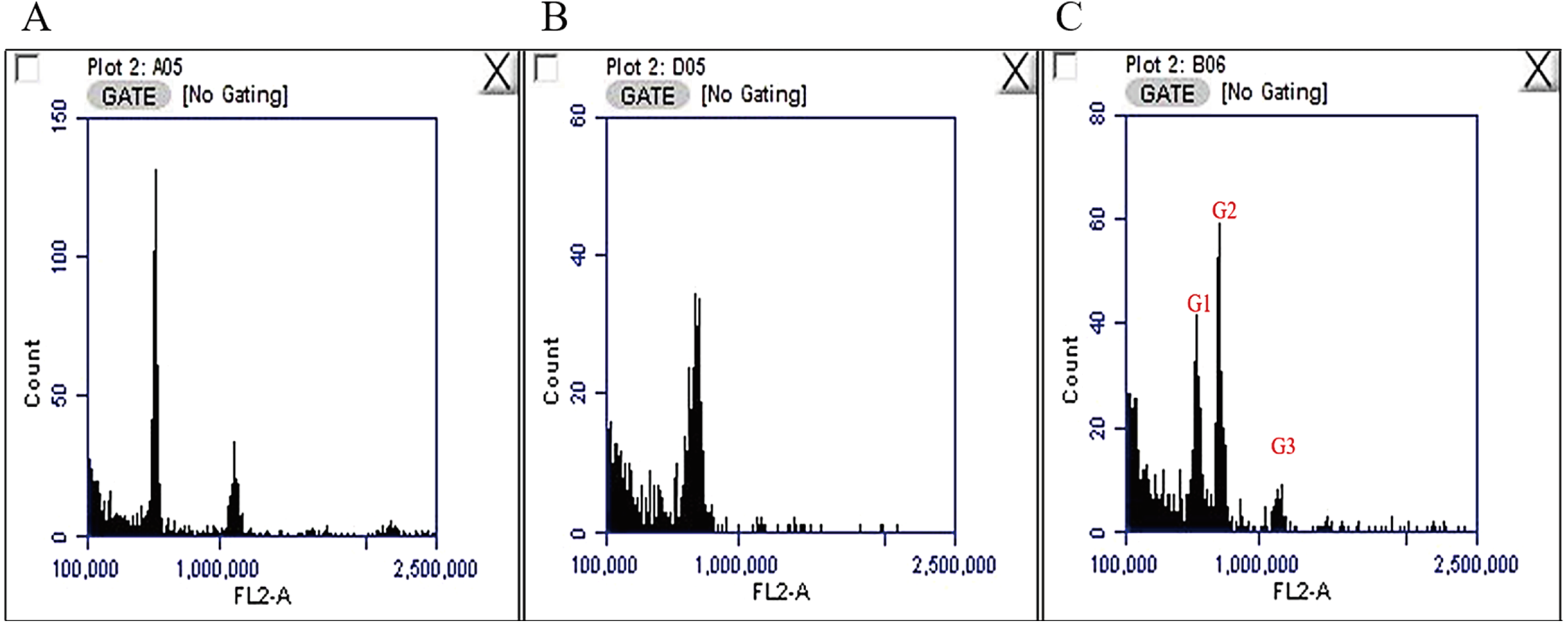
Fluorescence histograms for genome size assessments in *Cissus quadrangularis* by flow cytometry. (A) Radish (2C = 1.1 pg.) with peaks at about 585000 and 1146000, (B) *Cissus quadrangularis* with a peak at about 719000, (C) *Cissus quadrangularis* combined with Radish; G1—radish 2C peak, G2—*Cissus quadrangularis* 2C peak, G3—radish 4C peak.

The nuclear DNA content of *C. quadrangularis* was evaluated by flow cytometry using radish (2C = 1.11 pg) as the reference standard. The genome size for the *C. quadrangularis* individual considered in this study was estimated as 2C = 1.410 pg. (Fig. 1) representing 689 Mbp/1C (1 pg. = 978 Mbps) (Dolezel et al., 2003). According to the classification by Soltis et al. (2003) the *C. quadrangularis* genome falls within the group of plants with very small genome. The size of the *Cissus quadrangularis* genome is approximately in the same range as *Cissus antarctica* and *Cissus trifoliata* which are tetraploid species (Chu et al., 2018). In addition, its genome is roughly double that of *Cissus javana* and *Cissus rotundifolia* which have diploid genomes (Chu et al., 2018).

The mucilage of the chopped plant tissues had a viscous texture and is composed of complex polysaccharides of various concentrations, which include galacturonic acids, rhamnose, galactose and others (Ovodov, 1998). Their sticky nature causes the aggregation of the nuclei making it difficult to isolate them for cytometric analysis and in majority of species, leaves are commonly used for flow cytometry analysis. However, in our experiment, young leaves yielded unsatisfying results, which may have been caused by high amounts of polysaccharides. Following the suggestion by Suda (2004), petioles were used yielding acceptable peaks. Nuclei isolation buffers, including the Tris⋅MgCl_2_ buffer and Galbraiths’ buffer (Dolezel, Greilhuber & Suda, 2007), yielded unsatisfactory results (data not shown). Loureiro et al. (2006) modified the constituents of the Tris⋅MgCl_2_ buffer to develop WPB, which counters the negative effects of tannic acid. The inclusion of sodium metabisulfite and PVP-10 in the buffer enhanced its efficacy by reducing the impact of phenol and other secondary metabolites. Dolezel, Greilhuber & Suda (2007), Loureiro et al. (2007a) and Loureiro et al. (2007b) used higher concentrations of a detergent (Triton-X), which had the effect of reducing the mucilage viscosity levels and minimizing their negative impacts. WPB has been employed in genome size determination for other members in genus the *Cissus* genus (Chu et al., 2018) and other stubborn woody plants (Loureiro et al., 2007a; Loureiro et al., 2007b). Dark-treatment of the samples improved our results and is a method that can be applied in genome size determination for species with higher levels of polysaccharides and other secondary metabolites.

### Chromosome counts

**Figure 2:**
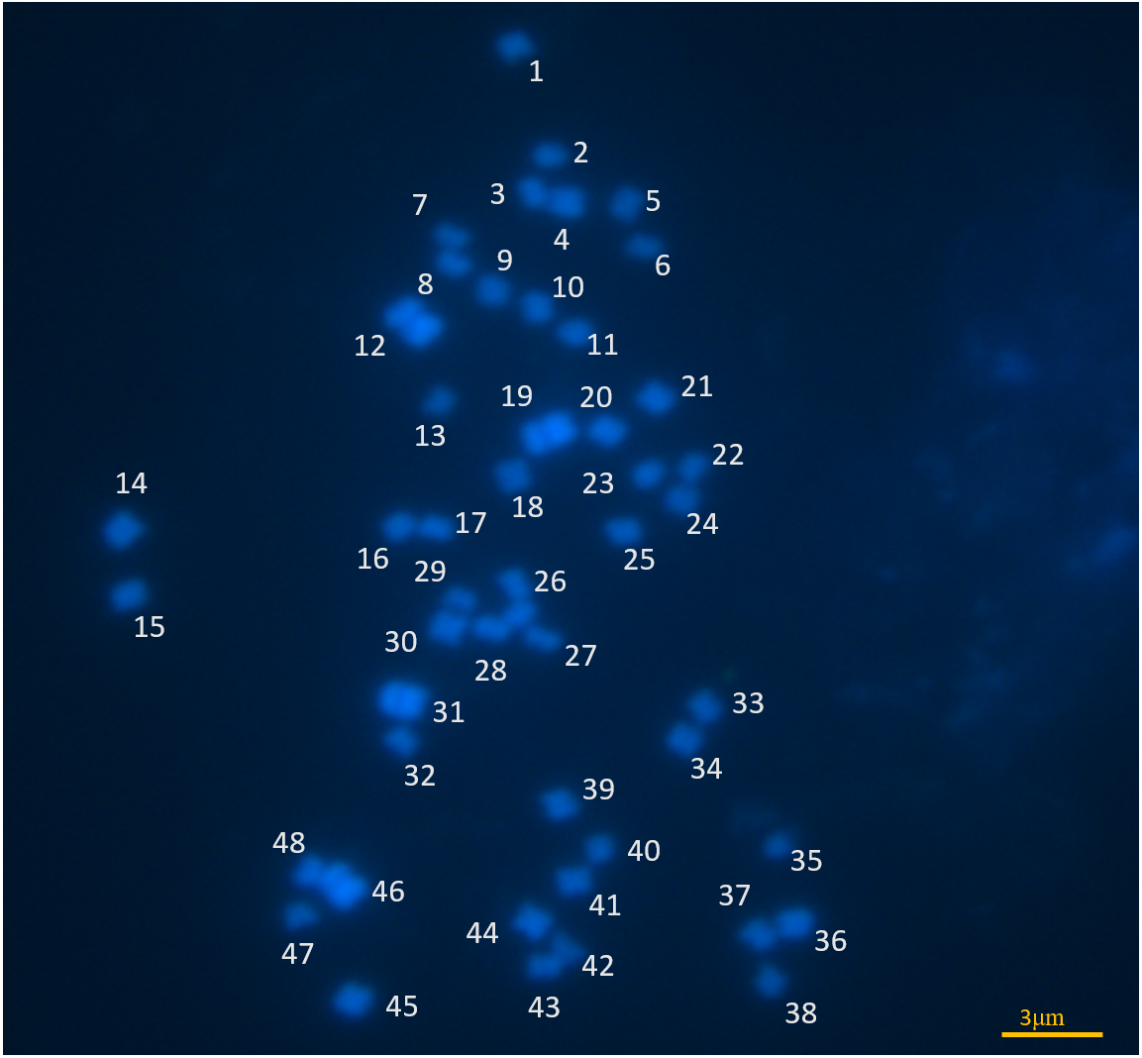
*Cissus quadrangularis* chromosome image. Mitotic metaphase chromosome complements from *Cissus quandrangularis*. The image has been resized for easy counting. Bar = 3 um.

The slides prepared with root tips were stained with a fluorescent dye, DAPI, to visualize the chromosomes. *C. quadrangularis* chromosomes are tiny and their numbers were quantified as 2*n* = 48 (Fig. 2). Additional chromosomal images have been supplied in Data S1. Observations revealed small chromosomes, making it difficult to identify the centromeres except for two pairs. To confirm the chromosome numbers, fluorescent *in situ* hybridization (FISH) using telomere labeled DNA probes was conducted. We used nick translation method to label DNA telomere probes (Ma et al., 2010). The sensitivity of FISH is to detect 3.5 kb target sequence on chromosome (Ma et al., 2010). Additionally, we used barley as the control for this experiment. We could not obtain clear telomere signals on all chromosome ends simultaneously in *Cissus quadrangularis*, while the telomere signals were clear in all barley chromosomes (data not shown). During FISH procedure, the DNA on the chromosomes could be lost/degraded if the fixation step is not optimized (Schwarzacher & Heslop-Harrison, 2000). In addition, high efficiencies FISH was achieved from mitotic metaphase chromosomes prepared from floral tissues of *Arabidopsis thaliana*, while almost no signals were detected on the chromosomes of root meristematic tissues with the same clones as probes. This is possibly due to the differences in chromatin structure between root meristematic tissues and floral tissues (Murata & Motoyoshi, 1995). In future, optimization of FISH protocol and use of different tissues to prepare mitotic metaphase chromosomes is therefore essential.

The basic chromosome number for members in the *Cissus* genus can be inferred to be 10, 11, or 12 (Chu et al., 2018). *Cissus quadrangularis* chromosomes have previously been reported (2*n* = 24, 28) for diploids and 2*n* = 45 for a specimen from India which was thought to be a tetraploid (Raghavan, 1957). Variations in chromosome numbers observed in individuals from different localities could be due to epigenetic factors such as DNA methylation and histone modification, which are thought to contribute to the silencing of transposable elements triggering chromosomal evolution in different biogeographical regions (Li et al., 2017). The limited sampling size prohibits a conclusive determination of the chromosome numbers for *C. quadrangularis.* In future studies, the sample size could be expanded to include more samples from different biogeographic locations.

Based on the deduced chromosome duplication model in the *Cissus* genus, previous reports for *C. quadrangularis* and other members in the genus (Karkamkar, Patil & Misra, 2010; http://www.tropicos.org/Project/IPCN; Chu et al., 2018), *C. quadrangularis* individual considered in our study, with 2*n* = 48 can be said to be a tetraploid (Table 1). However, more information, which may include molecular data, will be required to confirm this assumption. This would include carrying out both meiotic and mitotic studies to test the possibility of having accessory chromosomes, the existence of aneusomatic division, or the presence of accessory chromosomes in roots (Gibbs & Semir, 1988). Obtaining such information will improve our understanding of chromosomal evolution in the *Cissus* genus. This will further assist in the interpretation of phylogenetic and biogeographic models such as Cladistic, Migrationist and Panbiogeography models (Wilson, 1991), rather than just relying on potentially out-of-date information. Differences in diploid somatic chromosomes may be indicative of intraspecific polyploidization (Husband, Baldwin & Suda, 2013). Additionally, the occurrence of species complexes with variable karyotypes have been reported in other species and it has been suggested that they are indicative of a series of dysploidy in diploid species followed by hybridization and polyploidization (Choi et al., 2008; Haga & Noda, 1976). Such species complexes could suggest the presence of cryptic lineages in this species and therefore more tests should be carried out to include morphological traits examinations and molecular phylogenetics.

### The genome composition of *C. quadrangularis*

The complexity and tediousness of traditional molecular techniques have made it difficult to analyze genome components, especially in non-model organisms. In this study, short-read sequencing in combination with bioinformatics tools were used to analyze the *C. quadrangularis* genome. Genome analysis is efficient and economical using these techniques, despite the plant not being well-studied.

**Figure 3:**
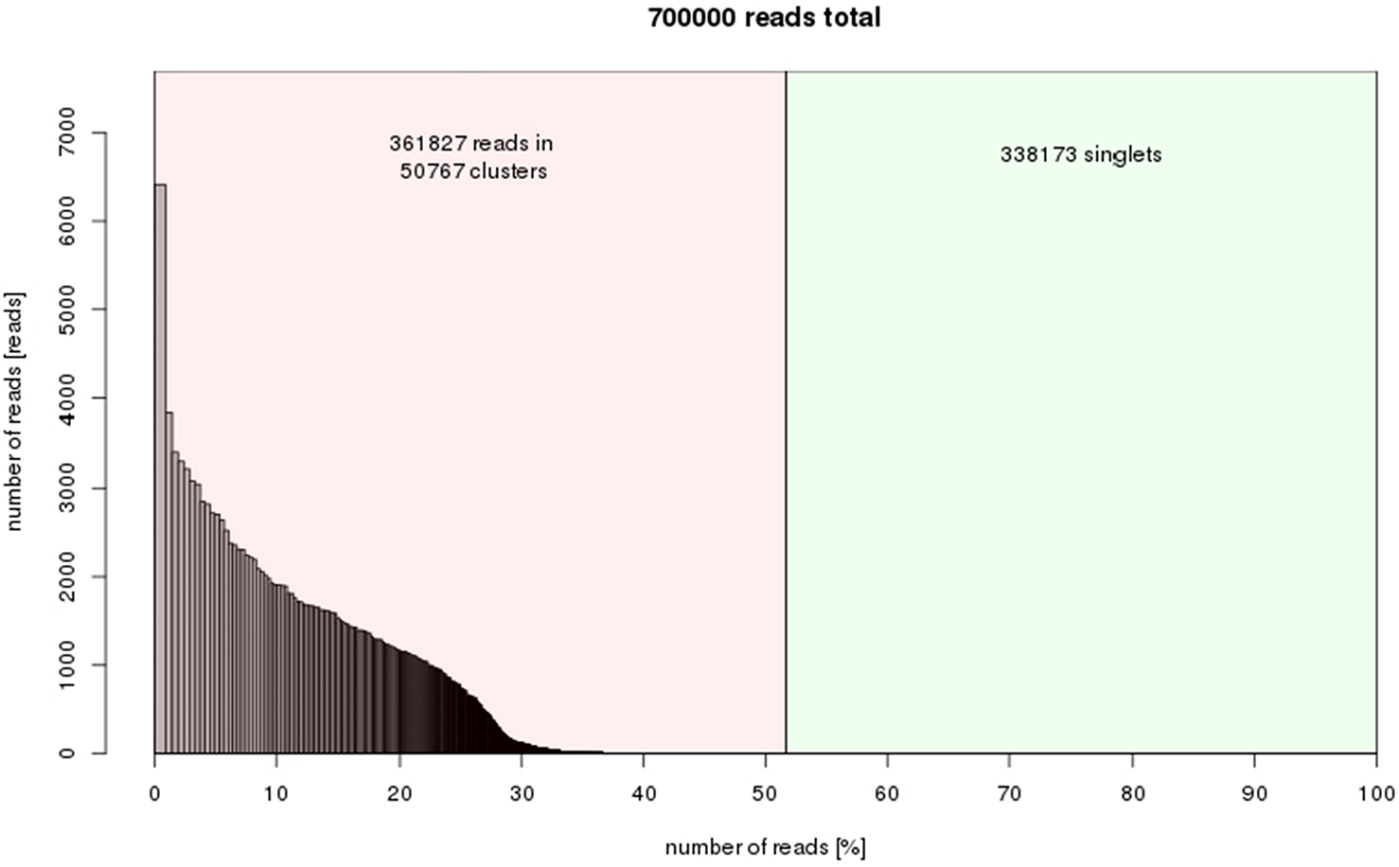
Repeat composition of clusters generated in RepeatExplorer (similarity-based partitioning) of 700000 reads (7.61% of genome size). *X*-axis: cumulative proportion of clusters of the genome. *Y*-axis: numbers of reads.

50,767 clusters were obtained from the RepeatExplorer results indicating that about 52% of the *C. quadrangularis* genome is composed of repetitive elements (Fig. 3). The output of clustering analysis forms a foundation for comprehensive study in the future to determine the repeat family structures and their variations. The raw RepeatExplorer output data has been provided in Data S2. The repeat composition was within the range of the estimates for plants with small genome sizes, which range between 25.04–66.42% in the *Oryza* genus (Zuccolo et al., 2007), 41.4% in grapes (Jaillon et al., 2007) and 61% in sorghum (Paterson et al., 2009).

The top 324 clusters constituting no less than 0.01% of the genome proportions were considered for further annotation. The relatively higher proportions of the single or low-copy repeat families were not considered. However, the variation of these elements may impact the phenotypic characteristics of plants (Barghini et al., 2015). To analyze the variations of the single copy retroelements, a reference genome in combination with genotype re-sequencing is needed, as in many model species. The singletons in this study represent the low-copy fraction of the genome that could not be assembled into clusters using RepeatExplorer (Fig. 3).

### Characterization of LTR-retrotransposons of *Cissus quadrangularis*

**Figure 4:**
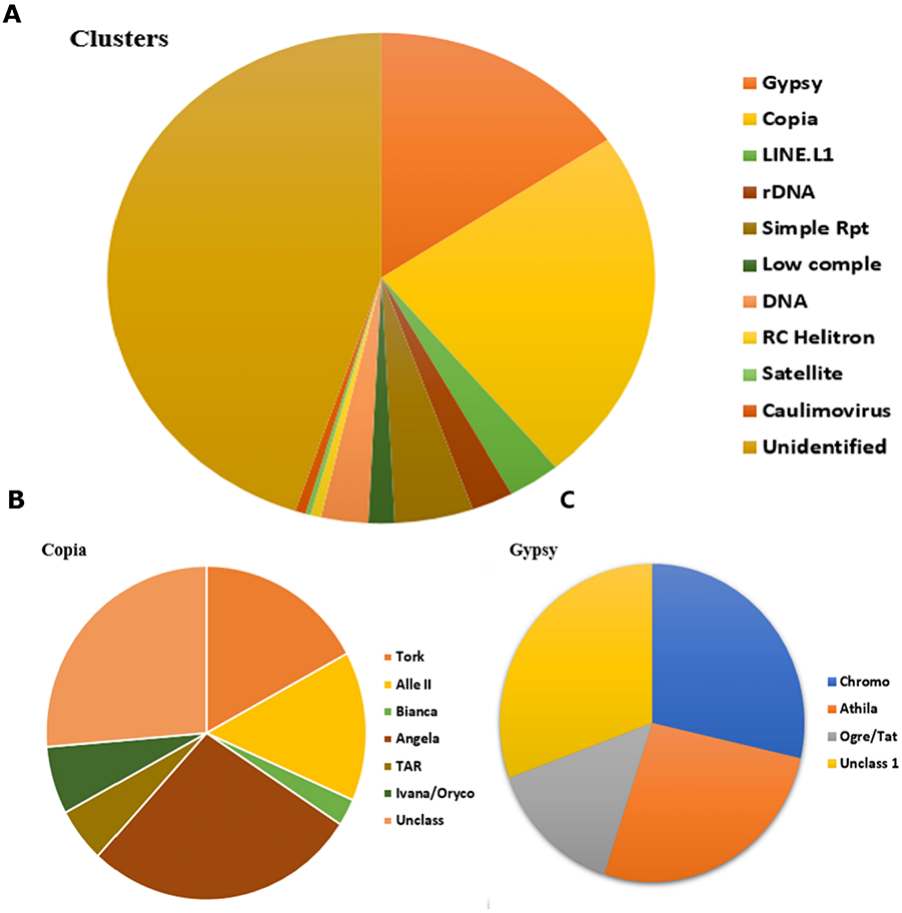
(A) The repeat class distribution of the 342 top clusters with no less than 0.01% of genome proportion from Illumina assemblies using RepeatExplorer, (B) & (C) lineage distributions of the main super-families, Copia and Gypsy, respectively. The values are percentages for each of the lineage composition.

Repeat cluster annotation and characterization were performed by a sequence similarities search against the NCBI database (Altschul et al., 1990) and on the RepeatExplorer platform for clusters with known protein domains. LTR-retrotransposons formed the major part of the identified repeats with two subfamilies: Gypsy and Copia. The Copia family comprised 24% of the LTR components while Gypsy made up of 15% of the LTR components (Fig. 4A). According to Wicker et al. (2007), the two main superfamilies, Gypsy and Copia, can further be classified into 6 and 7 lineages, respectively. The majority of the Copia lineages are of Angela type (Fig. 4B) (28% of the Copia elements) while the majority of the Gypsy lineages are of the chromovirus type (Fig. 4C) (29% of the Gypsy elements). A higher percentage of the analyzed clusters had unidentified components that represented clusters whose components could not be annotated (Fig. 4A). This is attributed to absence of protein domains in clusters and/or the limited repeat sequences of closely related species in public databases. Considerably higher numbers of unidentified clusters have been observed in the sunflower and *Eragrostis tef* (Gebre et al., 2016; Mascagni et al., 2015), while in sea grass (*Posidonia oceanica*) (Barghini et al., 2015), the proportions of unidentified clusters were considerably lower. Small clusters that were not analyzed (<0.01% genome proportion) also represented a significant proportion of the genome that was not well-characterized. Other components that were identified included LINEs, DNA transposons, organellar DNA, satellite repeats, and rDNA among others (Fig. 4A).

The possibility of chloroplast DNA insertions into the nuclear genome has been infrequently reported (Kejnovsky et al., 2006). In maize, the presence of chloroplast DNA in the nuclear genome has been reported (Roark et al., 2010), which may explain the presence of some levels of organellar DNA in our sample, while the majority could have been as a result of contamination during DNA extraction. The presence of rDNA can be due to biases generated during sequencing (Macas et al., 2011). Low efficiency during sequencing has been identified in GC-enriched segments when using Illumina technology (Nakamura et al., 2011; Aird et al., 2011) and leads to vulnerability of GC-rich templates to biases during Illumina sequencing. It is likely that the quality of the sequencing sample caused the bias in the representation of rDNA.

In this study, only 6 of the Copia subfamilies were found, which may be due to the limited consideration of clusters whose genome compositions were no less than 0.01% each were considered and therefore a large number of elements with lower representations were not annotated. In addition, part of the plant genome is not yet annotated into the main subfamilies as inferred from the analysis by RepeatExplorer. Therefore, more extensive sequencing techniques and coverage are required to decisively annotate the whole genome. We carried out repeat composition comparison analysis for *C. quadrangularis* and other well-studied species. From our comparison, a linear relationship between the genome size and LTR contents was observed but with exceptions (Table 3). For example, potato (*Solanum tuberosum*) has a genome size smaller than *C. quadrangularis* but a higher LTR composition. Our observations are in agreement with Wang et al. (2014) and Li et al. (2017) who identified a positive correlation between genome size and quantity of repetitive sequences in plants.

**Table 3:** Comparison of *Cissus quadrangularis* repeat composition with other studied plant species.

**Species**	**Genome size**	**LTR (%)**	**References**
Norway spruce	20 Gbps	60	Schnabel et al. (2009)
*Hordeum vulgare L*	4,289 Mbp	75	Nystedt et al. (2013)
*Zea may*	2,300 Mbp	70.10	Mayer et al. (2012)
*Solanum lycopersicum*	900 Mbp	61.8	Sato et al. (2012)
*Solanum tuberosum*	844 Mbp	29	Xu et al. (2011)
*Cissus quadrangularis*	689 Mbp	40	This study
*Actinidia chinensis*	616.1 Mbp	13.36	Huang et al. (2013)
*Vitis vinifera*	478 Mbp	6.30	Jaillon et al. (2007)

Variation in genome sizes for angiosperms have been attributed to differences in transposable element content, especially the LTR (Feng et al., 2017). This has been demonstrated in *Spirodela polyrhiza* (158 Mbp) with chromosome numbers 2*n* = 40 and *Lemma minor* which has the same chromosome number but a 481 Mbp genome size (Feng et al., 2017). Genomic repeats serve as vital indices in phylogenetic studies (Dodsworth et al., 2015). Therefore, changes in the LTR composition and its impact on genome evolution could be applied in evolutionary studies for *C. quadrangularis* and its relatives as new technologies are developed.

### Comparing the repeat contents for *Cissus quadrangularis*, grape, and 4 other *Cissus* species

The alignment of homologous DNA sequences has been the basis for molecular systematics. The differences in the sequence alignment patterns are used for the construction of phylogenetic trees. Insufficiency in divergences for homologous protein domains for repetitive elements between taxa makes repetitive elements unsuitable for phylogenetic analysis. However, variations in the number of repeat types and specific retrotransposons can be used quantitatively for phylogeny reconstruction (Dodsworth et al., 2015). Distantly related species exhibit divergences in the structure of their repetitive elements, whereas there is a level of uniformity noted in closely related species (Dodsworth et al., 2015).

**Figure 5:**
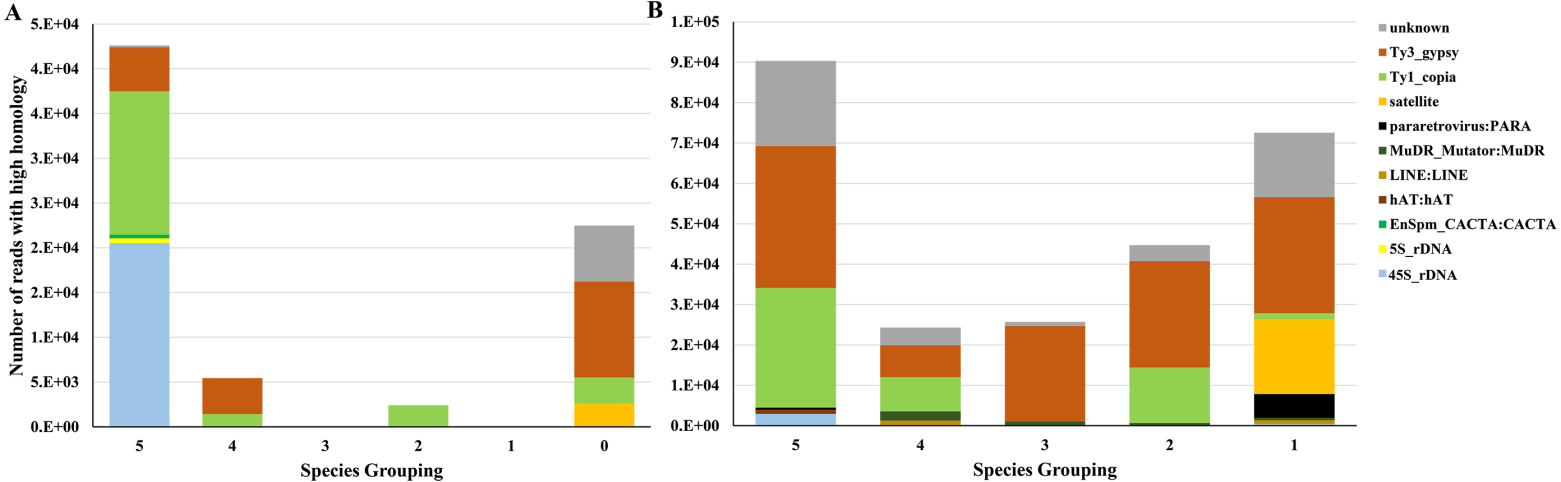
Comparison of transposable elements (TEs) for different *Cissus* species and grape. The numbers in *X*-axis represent the number of species combinations randomly selected. Different colors in *Y*-axis display types of TEs while the heights of the bars represent the number of reads. (A) The number of reads with high homology between *Cissus* species and grape. For example, the column ‘5’ represent the reads of different TEs in five *Cissus species* and grape, and the column ‘0’ represent the reads of different TEs unique to grape. (B) The number of reads with high homology across *Cissus* species without grape. For example, the column ‘5’ represent the reads of different TEs in five *Cissus* species (all *Cissus*), and column ‘1’ represent the reads of different TEs in one *Cissus*.

Based on our comparison, 45S rDNA was the major element present in all 6 species (Fig. 5A) which agreed with the high homology of 45S rDNA in angiosperm (Roa & Guerra, 2012). The application of repeat elements for phylogenetic inferences is defined up to the genus level due to a limited number of common repeats beyond genus level (Dodsworth et al., 2016). Therefore, phylogenetic inferences were restrained using repeats from the *Cissus* genus and grape. Other major elements represented in all of the six species studies include 5S rDNA, Ty_copia, Ty3_gypsy, and some elements whose protein domains could not be identified in the annotated clusters and which are identified as unknown in Figs. 5A & 5B. It was further noted that the most abundant repeats were shared by the 5 *Cissus* species (Fig. 5B). These elements include Ty3_gypsy, Ty1_copia, 45S rDNA, hAt:hAt, pararetrovirus:PARA and elements whose protein domains could not be identified (Fig. 5B). The noticeable conservation of the Copia elements may explain their homology for the species considered. Gypsy elements were shared among some species but the degree was lower compared to Copia elements. This indicates the less conservative nature of these elements as observed by Barghini et al. (2015). Other elements such as satellites and LINE: LINE were shared by some *Cissus* species, which may indicate that they are newly formed. These elements display a lower uniformity in the considered species and reflect variations in proliferation rates among different and related species (Hawkins et al., 2006).

Comparative analysis conducted on Musaceae family indicated quantitative differences in the repeat elements and the classified elements at different taxonomic levels (Novak et al., 2014). In our comparison, as a result of limited homology for majority of the repeat elements in *Cissus*, it was not possible to infer phylogenetic relationships. Dodsworth et al. (2015) noted a similar problem in constructing bifurcating phylogenetic trees while evaluating relationships in legume tribe Fabeae with homoploid and polyploid hybridization. In addition, comparative analysis of repetitive elements involving several species have unveiled variation in the sequences for probed repeat families and their abundances (Kelly et al., 2015; Macas et al., 2015). Novak et al. (2014) deduced that these differences may be due to the incomplete assembly and the unclear constituents that are encountered in the assembly of the genome. Considering the huge number of species in the *Cissus* genus, our sample size for co-clustering could contribute to the limited phylogenetic information in this genus. However, the information from this study is a foundation for future work in this genus.

## Conclusion

In this study, the genomic characteristics have been identified in the widely used species of the *Cissus* genus, *C. quadrangularis*. The high proportions of repetitive elements observed in *C. quadrangularis* could suggests that the expanded genome arose through the amplification of the repetitive elements coupled with polyploidization. Transposable element activities such as silencing and proliferation may have facilitated the observed karyotypic variations in this genus.

The probability of *C. quadrangularis* possessing a tetraploid genome has been implied based on the genome size expansion and the increase in chromosome numbers. However, more studies should be carried out to confirm the ploidy type. The information obtained in this study forms a foundation upon which additional phylogenetic and evolutionary studies can be carried out.

##  Supplemental Information

10.7717/peerj.8201/supp-1 Data S1 Additional Cissus quadrangularis chromosome images Additional Mitotic metaphase chromosomes compliments from *Cissus quandrangularis*

10.7717/peerj.8201/supp-2 Data S2 RepeatExplorer output data The data obtained from the pairwise comparison of short-read sequences are used to construct graphs with vertices representing the sequence reads and overlapping reads connected with edges. The edge weight is used to express the similarity scores for reads. Where the sequence genome coverage is low (<0.5 ×), presence of single copy sequences is low and therefore low overlapping resulting in detached nodes with no connections to other parts of the graph. Presence of repetitive sequences is represented by mutually connected nodes as a result of pooling of overlapping reads. Examination of graph topology facilitates separation and identification of clusters of reads frequently connected representing individual repetitive element families.

## Additional Information and Declarations

### DNA Deposition

The following information was supplied regarding the deposition of DNA sequences: Raw Illumina reads of Cissus quandrangularis are available at Figshare: Gichuki, Duncan Kiragu; Ma, Lu; Zhu, Zhenfei; Du, Chang; Hu, Guangwan; Zhong, Zhixiang; et al. (2019): Characters of repetitive elements in *Cissus quadrangularis* R1. figshare. Dataset. 10.6084/m9.figshare.7712219.v1. Ma, Lu; Gichuki, Duncan Kiragu; Zhu, Zhenfei; Du, Chang; Hu, Guangwan; Zhong, Zhixiang; et al. (2019): Characters of repetitive elements in *Cissus quadrangularis* R2. figshare. Dataset. 10.6084/m9.figshare.7712222.v1. Gichuki, Duncan Kiragu; Ma, Lu; Zhu, Zhenfei; Du, Chang; Hu, Guangwan; Zhong, Zhixiang; et al. (2019): RepeatExplorer output of Cissus quandrangularis. figshare. Dataset. 10.6084/m9.figshare.8115695.v1. Grape reads are available at SRA: SRR5627797. *Cissus discolor* reads are available at SRA: SRX1323033. *Cissus microcarpa* reads are available at SRA: SRX1322892. *Cissus trifoliata* reads are available at SRA: SRX1322890. *Cissus tuberosa* reads are available at SRA: SRX1322889.

### Data Availability

The following information was supplied regarding data availability: *Cissus quadrangularis* raw sequencing data are available at SRA: SRR8573652.
