# Supplementary figures and images for: Genome size, chromosome number determination, and analysis of the repetitive elements in Cissus quadrangularis
Source: PeerJ. 2019 Dec 20;7:e8201. doi: 10.7717/peerj.8201 (PMC6927348; doi:10.7717/peerj.8201)

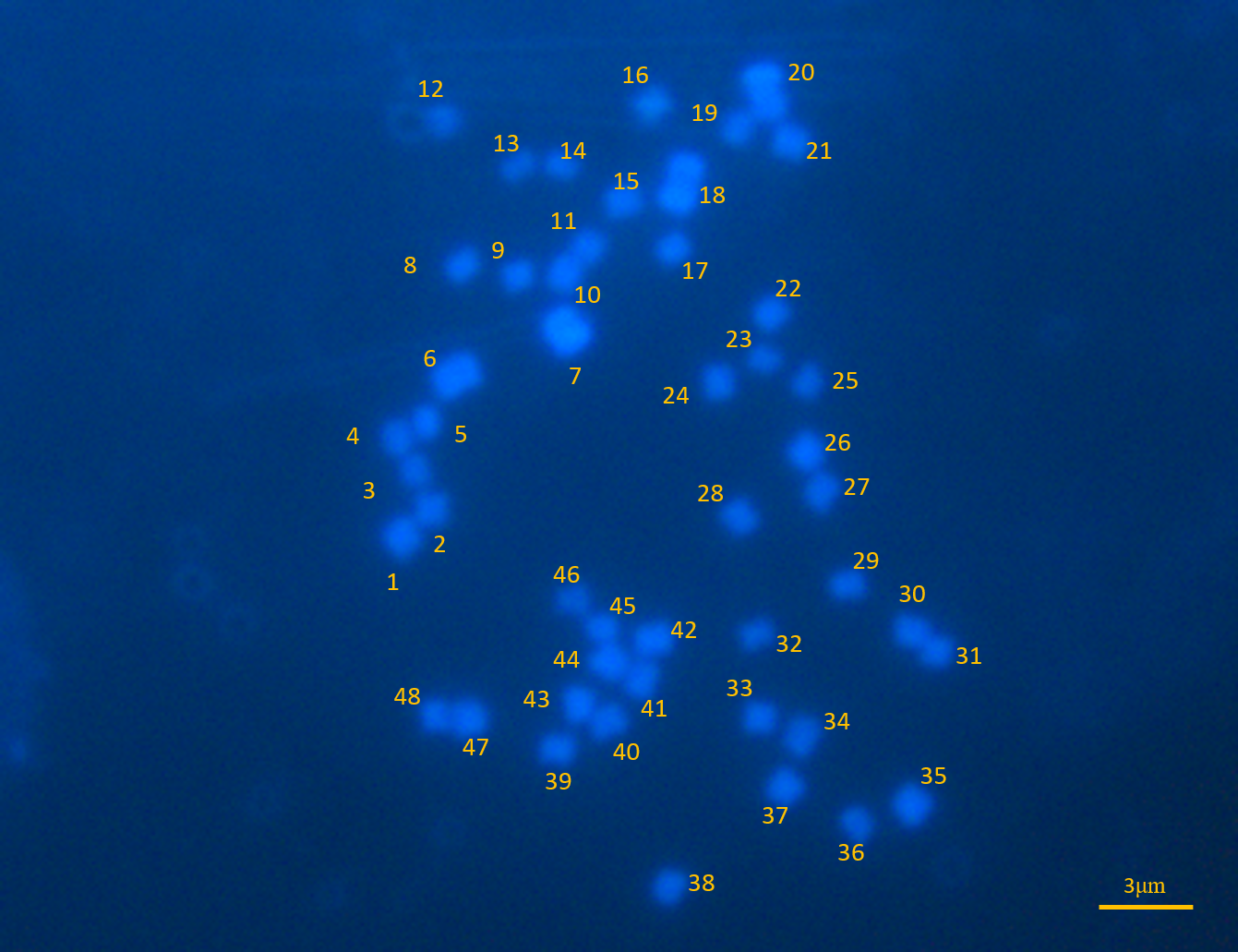

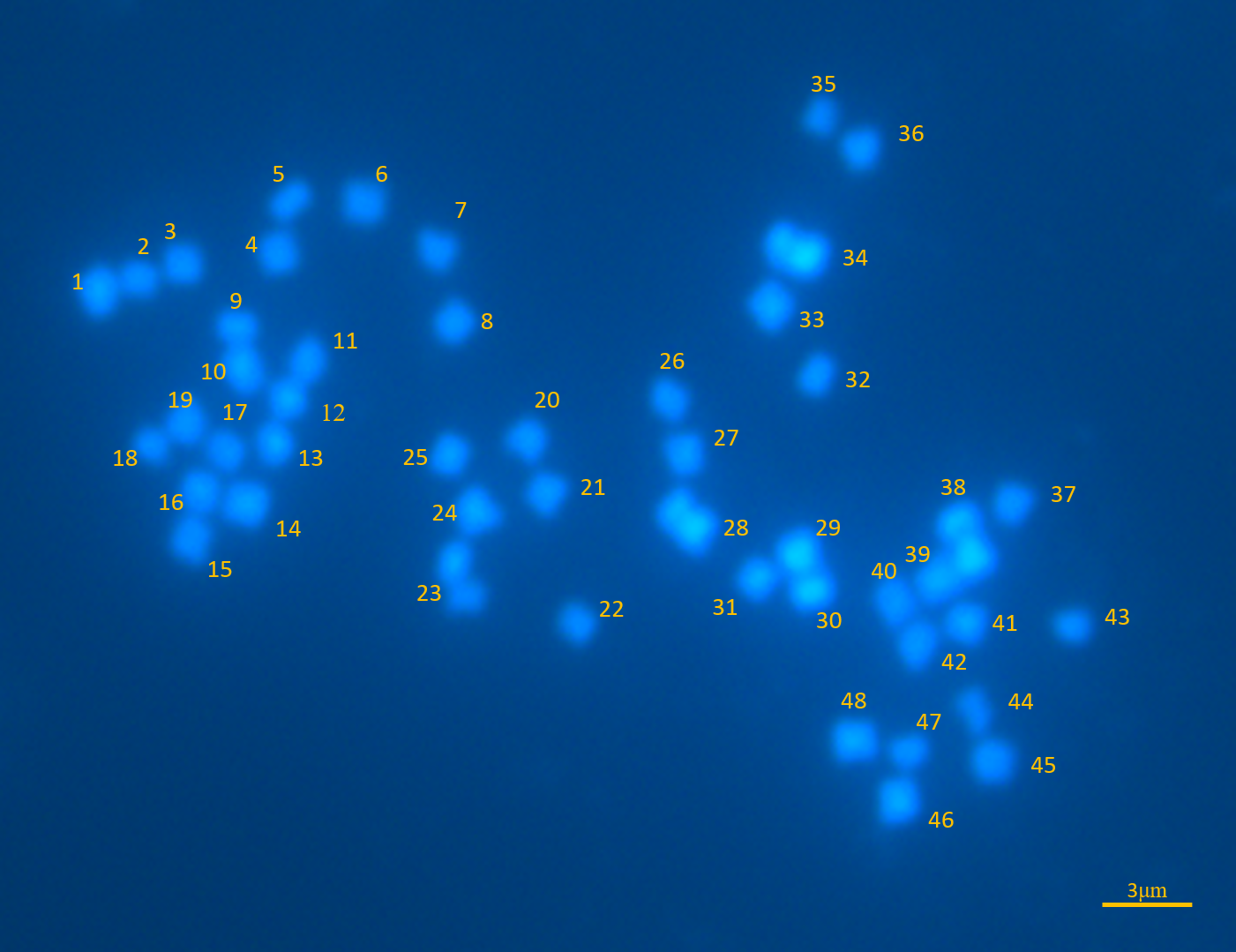

Supplement: Data S1 — Additional Mitotic metaphase chromosomes compliments from Cissus quandrangularis [file peerj-07-8201-s001.docx]
