# Supplementary material for: Genome size, chromosome number determination, and analysis of the repetitive elements in Cissus quadrangularis
Source: PeerJ. 2019 Dec 20;7:e8201. doi: 10.7717/peerj.8201 (PMC6927348; doi:10.7717/peerj.8201)
Supplement: Data S2 — The data obtained from the pairwise comparison of short-read sequences are used to construct graphs with vertices representing the sequence reads and overlapping reads connected with edges. The edge weight is used to express the similarity scores for reads. Where the sequence genome coverage is low (<0.5 ×), presence of single copy sequences is low and therefore low overlapping resulting in detached nodes with no connections to other parts of the graph. Presence of repetitive sequences is represented by mutually connected nodes as a result of pooling of overlapping reads. Examination of graph topology facilitates separation and identification of clusters of reads frequently connected representing individual repetitive element families. [file peerj-07-8201-s002.pdf]

Clustering summary

If you use RepeatExplorer in your work please cite:

Novak, P., Neumann, P., Pech, J., Steinhaisl, J., Macas, J. (2013) - RepeatExplorer: a Galaxy-based web server for genome-wide characterization of eukaryotic repetitive elements from next generation sequence reads. ***Bioinformatics*** **29:792-793.**
or
Novak, P., Neumann, P., Macas, J. (2010) - Graph-based clustering and characterization of repetitive sequences in next-generation sequencing data. ***BMC Bioinformatics*** **11:378.**

Sequence clustering results

Number of sequences used for clustering: 7e+05

Number of similarity hits: 7516066

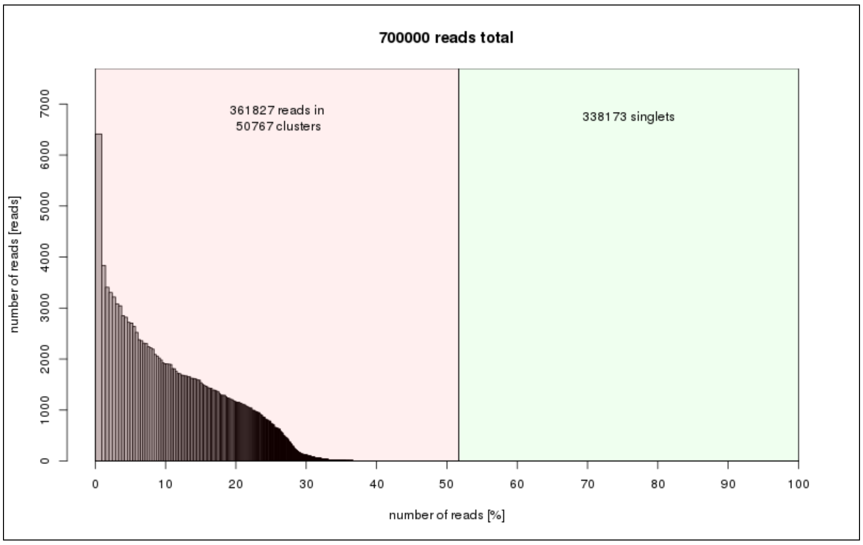

Disclaimer

Automatic classification is experimental feature and work in progress and the results should be used with caution!

Top clusters

	cluster	total length [bp]	number of reads	Genome proportion[%]	cumulative GP [%]	Automatic classification	Super cluster	Repeat Masker	Protein domain hits	blastn hits	Layout	Portion of similarity hits to other clusters[%]	Outside reads with similarity [%]
1	CL1	961950	6413	0.916	0.92	Not available	Not available	LTR.Gypsy (277hits, 2.71%)<br>Simple_repeat (141hits, 0.65%)<br>Low_complexity (81hits, 0.38%)<br>LTR.Copia (4hits, 0.0211%)<br>DNA.MULE.MuDR (1hits, 0.0078%)<br>DNA.hAT.Tip100 (1hits, 0.00468%)	DTA-CD1 NA NA (1 hits 0.0156%)<br>LINE-ENDO NA NA (1 hits 0.0156%)<br>LINE-RT NA NA (1 hits 0.0156%)<br>Ty3-RH Ty3/gypsy chromovirus (1 hits 0.0156%)<br>Ty3-RT Ty3/gypsy Athila (1 hits 0.0156%)		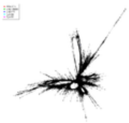	1.4850	5.380
2	CL2	575250	3835	0.548	1.46	Not available	Not available	Simple_repeat (300hits, 2.03%)<br>LTR.Gypsy (128hits, 1.78%)<br>Low_complexity (36hits, 0.3%)<br>DNA.CMC.EnSpm (3hits, 0.0389%)<br>DNA.MULE.MuDR (2hits, 0.0236%)<br>RC.Helitron (1hits, 0.0151%)<br>LTR.Copia (2hits, 0.015......	Ty1-RH Ty1/copia Tork (1 hits 0.0261%)		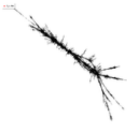	0.0187	0.157
3	CL3	511050	3407	0.487	1.95	Not available	Not available	LTR.Copia (**1644hits, 35.1%**)<br>LTR.Gypsy (30hits, 0.253%)<br>Simple_repeat (6hits, 0.0636%)<br>DNA.CMC.EnSpm (2hits, 0.0311%)<br>Low_complexity (2hits, 0.0188%)<br>DNA.P (1hits, 0.00939%)	DTC-CD1 NA NA (2 hits 0.0587%)<br>Ty3-INT Ty3/gypsy Athila (2 hits 0.0587%)<br>Ty1-INT Ty1/copia Tork (1 hits 0.0294%)<br>Ty3-GAG Ty3/gypsy Athila (1 hits 0.0294%)<br>Ty3-INT Ty3/gypsy Ogre/Tat (1 hits 0.0294%)		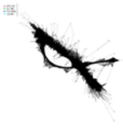	3.1540	13.710
4	CL4	495600	3304	0.472	2.42	Not available	Not available	LINE.L1 (**1789hits, 44.5%**)<br>Simple_repeat (18hits, 0.112%)<br>Low_complexity (7hits, 0.068%)<br>DNA.CMC.EnSpm (4hits, 0.0369%)<br>LTR.Copia (2hits,	LINE-RT NA NA (674 hits 20.4%)<br>LINE-ENDO NA NA (251 hits 7.6%)<br>DHH-CD1 NA NA (1 hits 0.0303%)		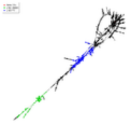	0.0000	0.000

								0.0254%)<br>Satellite (1hits, 0.0119%)					
5	CL5	482550	3217	0.460	2.88	Not available	Not available	LTR.Copia (**2331hits, 56.7%**)<br>Simple_repeat (4hits, 0.0317%)	Ty1-RT Ty1/copia Angela (2 hits 0.0622%)		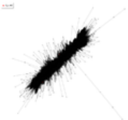	4.5200	20.920
6	CL6	462450	3083	0.440	3.32	Not available	Not available	LTR.Copia (**224hits, 3.43%**)<br>LTR.Gypsy (7hits, 0.0787%)<br>Simple_repeat (9hits, 0.0722%)<br>Low_complexity (2hits, 0.0195%)	Ty1-RT Ty1/copia AleII (2 hits 0.0649%)<br>DTM-CD1 NA NA (1 hits 0.0324%)<br>Ty1-INT Ty1/copia AleII (1 hits 0.0324%)<br>Ty1-INT Ty1/copia Angela (1 hits 0.0324%)<br>Ty1-RT Ty1/copia Maximus/SIRE (1 hits 0.0324%)<br>Ty3-......		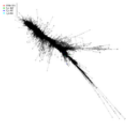	3.5370	10.740
7	CL7	456600	3044	0.435	3.76	Not available	Not available	Simple_repeat (57hits, 0.61%)<br>LTR.Gypsy (25hits, 0.299%)<br>Low_complexity (18hits, 0.191%)<br>LTR.Copia (1hits, 0.0129%)	Ty3-INT Ty3/gypsy Athila (1 hits 0.0329%)<br>Ty3-INT Ty3/gypsy Ogre/Tat (1 hits 0.0329%)	organelle/plastid (1 hits 0.0329%)	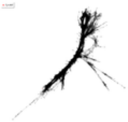	2.7140	16.330
8	CL8	427350	2849	0.407	4.16	Not available	Not available	LTR.Copia (**1410hits, 42.8%**)<br>Simple_repeat (126hits, 1.37%)<br>DNA.hAT.Ac (8hits, 0.17%)<br>Low_complexity (9hits, 0.155%)<br>LTR.Gypsy (4hits, 0.0644%)<br>Satellite (1hits, 0.00702%)	Ty1-RT Ty1/copia Bianca (424 hits 14.9%)<br>Ty1-RH Ty1/copia Bianca (215 hits 7.55%)<br>LINE-RT NA NA (1 hits 0.0351%)<br>Ty1-RT Ty1/copia AleI/Retrofit (1 hits 0.0351%)	organelle/mitochondria (1 hits 0.0351%)	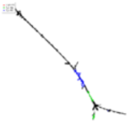	0.0046	0.035
9	CL9	423000	2820	0.403	4.57	Not available	Not available	LTR.Gypsy (**640hits, 15.5%**)<br>Simple_repeat (89hits, 0.681%)<br>Low_complexity (16hits, 0.213%)<br>LTR.Copia (1hits, 0.0229%)	DTC-CD1 NA NA (1 hits 0.0355%)<br>Ty3-RH Ty3/gypsy chromovirus (1 hits 0.0355%)<br>Ty3-RT Ty3/gypsy chromovirus (1 hits 0.0355%)		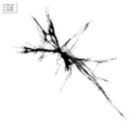	1.1710	9.043
10	CL10	407850	2719	0.388	4.96	Not available	Not available	LTR.Gypsy (**1999hits, 62.5%**)<br>LTR.Copia (36hits, 0.563%)<br>Simple_repeat (2hits, 0.0184%)<br>Low_complexity (1hits, 0.0108%)<br>rRNA (1hits, 0.00662%)	Ty3-INT Ty3/gypsy chromovirus (436 hits 16%)<br>Ty3-RT Ty3/gypsy chromovirus (304 hits 11.2%)<br>Ty3-GAG Ty3/gypsy chromovirus (220 hits 8.09%)<br>Ty3-RH Ty3/gypsy chromovirus (165 hits 6.07%)<br>Ty3-PROT Ty3/gypsy chrom......	organelle/mitochondria (15 hits 0.552%)	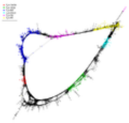	0.1122	0.515
11	CL11	405750	2705	0.386	5.34	Not available	Not available	LTR.Gypsy (**2713hits, 89%**)<br>LTR.Copia. (133hits, 1.8%)<br>Low_complexity (4hits, 0.0762%)<br>Simple_repeat (4hits, 0.0518%)<br>LTR (1hits, 0.0325%)	Ty3-INT Ty3/gypsy Athila (1228 hits 45.4%)<br>Ty3-RH Ty3/gypsy Athila (449 hits 16.6%)<br>Ty3-RT Ty3/gypsy Athila (167 hits 6.17%)<br>Ty3-GAG Ty3/gypsy chromovirus (1 hits 0.037%)<br>Ty3-INT Ty3/gypsy Ogre/Tat (1 hits 0.037%)	organelle/mitochondria (4 hits 0.148%)<br>organelle/plastid (1 hits 0.037%)	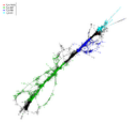	0.6020	2.625
12	CL12	396150	2641	0.377	5.72	Not available	Not available	Simple_repeat (48hits, 0.42%)<br>Low_complexity (12hits, 0.147%)<br>LTR.Gypsy (1hits, 0.0212%)<br>LTR.Copia. (2hits, 0.0202%)<br>DNA.CMC.EnSpm (2hits, 0.0187%)<br>DNA.MULE.MuDR (1hits, 0.0159%)			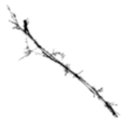	0.3460	1.250
13	CL13	378600	2524	0.361	6.08	Not available	Not available	LTR.Gypsy (107hits, 2.07%)<br>Low_complexity (64hits, 1.12%)<br>Simple_repeat (65hits, 0.875%)	Ty3-RT Ty3/gypsy Athila (2 hits 0.0792%)<br>Ty3-GAG Ty3/gypsy Athila (1 hits 0.0396%)<br>Ty3-INT Ty3/gypsy	organelle/mitochondria (1 hits 0.0396%)	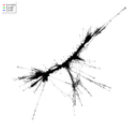	2.8120	22.820

							LTR.Copia (3hits, 0.0338%)	Athila (1 hits 0.0396%)				
14	CL14	356700 2378	0.340	6.42	Not available	Not available	LTR.Copia (**882hits, 27%**)<br>Simple_repeat (41hits, 0.513%)<br>LTR.Gypsy (2hits, 0.0252%)<br>DNA.CMC.EnSpm (1hits, 0.0154%)	Ty1-GAG Ty1/copia Angela (380 hits 16%)<br>DHH-CD2 NA NA (1 hits 0.0421%)<br>Ty1-INT Ty1/copia AleI/Retrofit (1 hits 0.0421%)		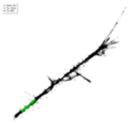	1.1670	3.911
15	CL15	353850 2359	0.337	6.76	Not available	Not available	LTR.Gypsy (**497hits, 14.1%**)<br>Simple_repeat (80hits, 0.832%)<br>Low_complexity (50hits, 0.707%)<br>LTR.Copia (15hits, 0.239%)<br>DNA.CMC.EnSpm (1hits, 0.0147%)	DTM-CD1 NA NA (2 hits 0.0848%)<br>Ty1-INT Ty1/copia Angela (2 hits 0.0848%)<br>Ty1-RT Ty1/copia Angela (1 hits 0.0424%)<br>Ty1-RT Ty1/copia Tork (1 hits 0.0424%)<br>Ty3-RH Ty3/gypsy chromovirus (1 hits 0.0424%)		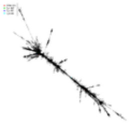	0.0663	0.466
16	CL16	346650 2311	0.330	7.09	Not available	Not available	LTR.Copia (**611hits, 17.1%**)<br>Simple_repeat (47hits, 0.637%)<br>LTR.Gypsy (16hits, 0.243%)	Ty3-INT Ty3/gypsy Ogre/Tat (7 hits 0.303%)<br>Ty1-RH Ty1/copia Angela (1 hits 0.0433%)		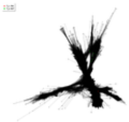	6.9220	20.770
17	CL17	345750 2305	0.329	7.42	Not available	Not available	LTR.Copia (**1625hits, 61.4%**)<br>Simple_repeat (49hits, 0.649%)<br>Low_complexity (10hits, 0.141%)<br>LTR.Gypsy (1hits, 0.0142%)	Ty1-INT Ty1/copia Angela (550 hits 23.9%)<br>Ty1-GAG Ty1/copia Angela (336 hits 14.6%)<br>Ty1-PROT Ty1/copia Angela (224 hits 9.72%)		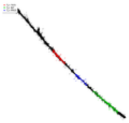	2.0640	8.416
18	CL18	336300 2242	0.320	7.74	Not available	Not available	LTR.Copia (**2200hits, 92%**)<br>LTR.Gypsy (2hits, 0.0274%)	Ty1-RT Ty1/copia Angela (1879 hits 83.8%)<br>Ty1-RT Ty1/copia AleI/Retrofit (1 hits 0.0446%)<br>Ty1-RT Ty1/copia AleII (1 hits 0.0446%)		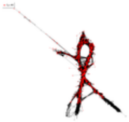	4.4030	12.310
19	CL19	332850 2219	0.317	8.05	Not available	Not available	rRNA (**961hits, 38.3%**)		45S_rDNA/18S_rDNA (937 hits 42.2%)<br>organelle/mitochondria (13 hits 0.586%)	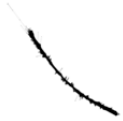	2.8550	8.157
20	CL20	329100 2194	0.313	8.37	Not available	Not available	LTR.Copia (**2116hits, 90.6%**)<br>LTR.Gypsy (2hits, 0.0249%)<br>LTR (1hits, 0.0188%)	Ty1-RH Ty1/copia Angela (1033 hits 47.1%)<br>Ty1-RT Ty1/copia Angela (208 hits 9.48%)		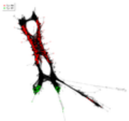	6.2240	17.180
21	CL21	313350 2089	0.298	8.67	Not available	Not available	Simple_repeat (104hits, 1.46%)<br>LTR.Copia (95hits, 0.893%)<br>LTR.Gypsy (3hits, 0.0654%)<br>rRNA (2hits, 0.0453%)<br>Low_complexity (3hits, 0.0329%)<br>DNA.MULE.MuDR. (1hits, 0.023%)<br>DNA.MULE.MuDR (1hits, 0.014%)	DTM-CD1 NA NA (1 hits 0.0479%)<br>Ty1-GAG Ty1/copia Bianca (1 hits 0.0479%)<br>Ty1-INT Ty1/copia Angela (1 hits 0.0479%)<br>Ty3-GAG Ty3/gypsy Athila (1 hits 0.0479%)	45S_rDNA/18S_rDNA (2 hits 0.0957%)	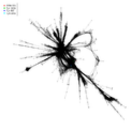	0.5733	5.026
22	CL22	308850 2059	0.294	8.96	Not available	Not available	LTR.Gypsy (**1575hits, 69.8%**)<br>Low_complexity (53hits, 1.07%)<br>Simple_repeat (50hits, 0.923%)<br>DNA.CMC.EnSpm (1hits, 0.0133%)	Ty3-RT Ty3/gypsy Athila (524 hits 25.4%)<br>Ty3-PROT Ty3/gypsy Athila (320 hits 15.5%)<br>Ty3-PROT Ty3/gypsy Ogre/Tat (7 hits 0.34%)<br>LINE-RT NA NA (1 hits 0.0486%)	organelle/plastid (4 hits 0.194%)	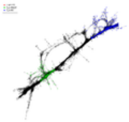	0.7264	3.788
23	CL23	303000 2020	0.289	9.25	Not available	Not available	LTR.Gypsy (**643hits, 18%**)<br>Low_complexity (30hits, 0.5%)<br>Simple_repeat (25hits, 0.383%)<br>DNA.PIF.Harbinger (5hits, 0.0604%)	Ty3-GAG Ty3/gypsy Athila (454 hits 22.5%)	organelle/mitochondria (1 hits 0.0495%)	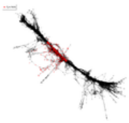	0.5129	2.624

								LINE.L1 (1hits, 0.0182%)<br>DNA.CMC.EnSpm (1hits, 0.0155%)					
24	CL24	297150	1981	0.283	9.53	Not available	Not available	LTR.Gypsy (**259hits, 8.37%**)<br>LTR.Copia (13hits, 0.536%)<br>Simple_repeat (31hits, 0.414%)<br>Low_complexity (22hits, 0.31%)<br>DNA.MULE.MuDR (1hits, 0.0158%)<br>tRNA (1hits, 0.0138%)	Ty1-RH Ty1/copia Ivana/Oryco (9 hits 0.454%)<br>DTM-CD1 NA NA (1 hits 0.0505%)<br>Ty1-RT Ty1/copia Angela (1 hits 0.0505%)<br>Ty1-RT Ty1/copia Ivana/Oryco (1 hits 0.0505%)	organelle/mitochondria (2 hits 0.101%)	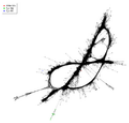	0.3186	2.625
25	CL25	288600	1924	0.275	9.81	Not available	Not available	LTR.Copia (**1576hits, 70.4%**)<br>Simple_repeat (9hits, 0.119%)<br>LTR.Gypsy (4hits, 0.0894%)<br>LTR.Caulimovirus (4hits, 0.087%)<br>LINE.L1 (2hits, 0.044%)<br>LTR (1hits, 0.0326%)<br>DNA.PIF.Harbinger (1hits, 0.0118%)	Ty1-INT Ty1/copia Tork (258 hits 13.4%)<br>Ty1-GAG Ty1/copia Tork (206 hits 10.7%)<br>Ty1-RT Ty1/copia Tork (158 hits 8.21%)<br>Ty1-PROT Ty1/copia Tork (107 hits 5.56%)<br>Ty1-RH Ty1/copia Tork (68 hits 3.53%)<br>DTM-CD1......	organelle/mitochondria (13 hits 0.676%)	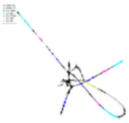	0.2428	0.676
26	CL26	286350	1909	0.273	10.10	Not available	Not available	LTR.Copia (**1739hits, 85.5%**)	Ty1-INT Ty1/copia Angela (934 hits 48.9%)<br>Ty1-PROT Ty1/copia Angela (325 hits 17%)<br>LINE-RT NA NA (1 hits 0.0524%)		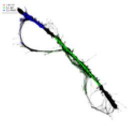	3.2140	12.410
27	CL27	285600	1904	0.272	10.40	Not available	Not available	Simple_repeat (109hits, 1.33%)<br>Low_complexity (30hits, 0.483%)<br>LTR.Copia (1hits, 0.0119%)		organelle/plastid (1975 hits 104%)<br>organelle/mitochondria (153 hits 8.04%)	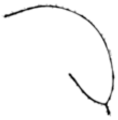	0.8263	3.624
28	CL28	285000	1900	0.271	10.60	Not available	Not available	rRNA (**1889hits, 96.9%**)<br>Simple_repeat (1hits, 0.0151%)		45S_rDNA/18S_rDNA (1881 hits 99%)<br>45S_rDNA/25S_rDNA (1 hits 0.0526%)	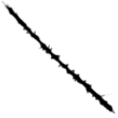	2.2690	4.579
29	CL29	284100	1894	0.271	10.90	Not available	Not available	Simple_repeat (101hits, 1.29%)<br>Low_complexity (27hits, 0.479%)<br>LTR.Copia (11hits, 0.367%)	Ty1-RH Ty1/copia AleI/Retrofit (10 hits 0.528%)	organelle/plastid (1490 hits 78.7%)<br>organelle/mitochondria (354 hits 18.7%)	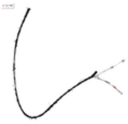	0.5818	2.218
30	CL30	271950	1813	0.259	11.20	Not available	Not available	DNA.CMC.EnSpm (90hits, 1.7%)<br>Simple_repeat (3hits, 0.068%)		organelle/plastid (3680 hits 203%)	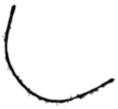	1.4290	5.681
31	CL31	271500	1810	0.259	11.40	Not available	Not available	LTR.Gypsy (89hits, 1.19%)<br>Simple_repeat (31hits, 0.542%)<br>Low_complexity (8hits, 0.149%)		organelle/plastid (3368 hits 186%)<br>organelle/mitochondria (1 hits 0.0552%)	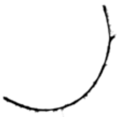	0.8964	4.420
32	CL32	263850	1759	0.251	11.70	Not available	Not available	LTR.Copia (**1029hits, 50.4%**)<br>Simple_repeat (31hits, 0.456%)<br>Low_complexity (22hits, 0.352%)<br>LTR.Gypsy (7hits, 0.121%)	Ty1-RT Ty1/copia TAR (157 hits 8.93%)<br>Ty1-RH Ty1/copia TAR (88 hits 5%)<br>Ty1-GAG Ty1/copia TAR (44 hits 2.5%)<br>Ty1-RT Ty1/copia AleII (7 hits 0.398%)<br>Ty1-RT Ty1/copia AleI/Retrofit (5 hits 0.284%)		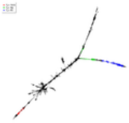	0.0408	0.341

33	CL33	258000	1720	0.246	11.90	Not available	Not available	LTR.Copia (**1549hits, 83.6%**)<br>Simple_repeat (32hits, 0.454%)<br>LINE.L1 (3hits, 0.13%)	Ty1-RH Ty1......<br>Ty1-RT Ty1/copia AleII (688 hits 40%)<br>Ty1-RH Ty1/copia AleII (404 hits 23.5%)<br>Ty1-RT Ty1/copia Ivana/Oryco (21 hits 1.22%)<br>LINE-RT NA NA (4 hits 0.233%)<br>Ty1-RT Ty1/copia Tork (4 hits 0.233%)<br>Ty1-RT Ty1/cop......	organelle/mitochondria (97 hits 5.64%)	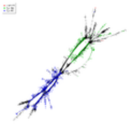	0.1617	0.930
34	CL34	257100	1714	0.245	12.20	Not available	Not available	Simple_repeat (189hits, 2.73%)<br>LTR.Copia (22hits, 0.499%)<br>DNA.CMC.EnSpm (39hits, 0.497%)	DTM-CD1 NA NA (3 hits 0.175%)<br>Ty1-RT Ty1/copia Ivana/Oryco (1 hits 0.0583%)	organelle/plastid (1608 hits 93.8%)<br>organelle/mitochondria (80 hits 4.67%)	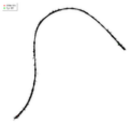	0.3056	1.109
35	CL35	253050	1687	0.241	12.40	Not available	Not available	Simple_repeat (68hits, 0.833%)<br>Low_complexity (13hits, 0.246%)<br>LTR.Gypsy (1hits, 0.0126%)		organelle/plastid (1585 hits 94%)<br>organelle/mitochondria (135 hits 8%)	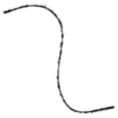	0.2489	1.897
36	CL36	252300	1682	0.240	12.60	Not available	Not available	Simple_repeat (126hits, 1.73%)<br>LTR.Copia (1hits, 0.019%)<br>Low_complexity (1hits, 0.0123%)		organelle/plastid (1568 hits 93.2%)<br>organelle/mitochondria (132 hits 7.85%)	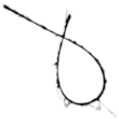	0.3549	1.962
37	CL37	250800	1672	0.239	12.90	Not available	Not available	LTR.Gypsy (**492hits, 20.6%**)<br>Simple_repeat (69hits, 0.953%)<br>Low_complexity (28hits, 0.638%)<br>LTR.Copia. (7hits, 0.141%)<br>LTR (1hits, 0.0459%)	DTM-CD1 NA NA (1 hits 0.0598%)<br>LINE-ENDO NA NA (1 hits 0.0598%)		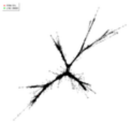	1.0030	7.177
38	CL38	250200	1668	0.238	13.10	Not available	Not available	Simple_repeat (89hits, 1.32%)<br>Low_complexity (1hits, 0.0136%)		organelle/plastid (3286 hits 197%)<br>organelle/mitochondria (36 hits 2.16%)	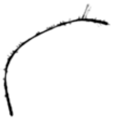	1.8510	5.156
39	CL39	248250	1655	0.236	13.30	Not available	Not available	LTR.Gypsy (**122hits, 4.06%**)<br>Simple_repeat (76hits, 1.57%)<br>Low_complexity (55hits, 1.5%)<br>LTR.Copia (11hits, 0.251%)	Ty1-INT Ty1/copia Ivana/Oryco (1 hits 0.0604%)<br>Ty1-RT Ty1/copia Angela (1 hits 0.0604%)<br>Ty3-RH Ty3/gypsy Athila (1 hits 0.0604%)		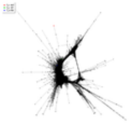	2.9970	14.800
40	CL40	247500	1650	0.236	13.60	Not available	Not available	LTR.Gypsy (**318hits, 12.6%**)<br>Simple_repeat (42hits, 0.768%)<br>Low_complexity (15hits, 0.339%)<br>LTR.Copia (8hits, 0.191%)	Ty3-RT Ty3/gypsy Athila (2 hits 0.121%)<br>Ty1-INT Ty1/copia AleII (1 hits 0.0606%)		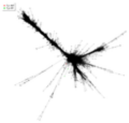	0.4266	5.455
41	CL41	243450	1623	0.232	13.80	Not available	Not available	Simple_repeat (89hits, 1.04%)		organelle/plastid (3235 hits 199%)<br>organelle/mitochondria (59 hits 3.64%)	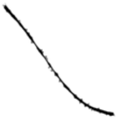	1.1920	4.559
42	CL42	243000	1620	0.231	14.00	Not available	Not available	rRNA (**146hits, 4.84%**)		organelle/plastid (3098 hits 191%)<br>organelle/mitochondria (82 hits 5.06%)	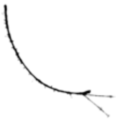	1.3820	3.333
								LTR.Gypsy (97hits, 1.93%)	Ty3-RH Ty3/gypsy Athila (4 hits 0.247%)<br>Ty1-RT Ty1/copia Angela (3 hits 0.185%)		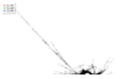		

43	CL43	242700	1618	0.231	14.30	Not available	Not available	LTR.Copia (8hits, 0.183%) Simple_repeat (7hits, 0.119%)	[illegible] Ty3-INT Ty3/gypsy Athila (2 hits 0.124%) Ty3-INT Ty3/gypsy chromovirus (2 hits 0.124%)		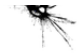	1.2150	7.169
44	CL44	240600	1604	0.229	14.50	Not available	Not available	rRNA (**1605hits, 98.5%**) Simple_repeat (1hits, 0.0121%)		45S_rDNA/25S_rDNA (1601 hits 99.8%)	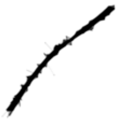	4.2430	11.160
45	CL45	238350	1589	0.227	14.70	Not available	Not available	Simple_repeat (35hits, 0.684%) LTR.Gypsy (7hits, 0.369%) Low_complexity (2hits, 0.0537%)			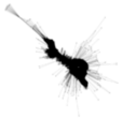	0.9682	10.510
46	CL46	237750	1585	0.226	15.00	Not available	Not available	Simple_repeat (83hits, 1.34%) Low_complexity (1hits, 0.0177%)		organelle/plastid (1571 hits 99.1%) organelle/mitochondria (1 hits 0.0631%)	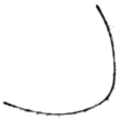	0.9126	2.587
47	CL47	230400	1536	0.219	15.20	Not available	Not available	rRNA (**1346hits, 81.8%**) Simple_repeat (71hits, 1.29%) Low_complexity (39hits, 0.557%)		45S_rDNA/25S_rDNA (1294 hits 84.2%)	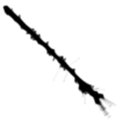	2.7320	6.380
48	CL48	225750	1505	0.215	15.40	Not available	Not available	LTR.Gypsy (**1401hits, 84.6%**) LTR.Copia. (11hits, 0.275%) Low_complexity (8hits, 0.205%) Simple_repeat (7hits, 0.13%)	Ty3-INT Ty3/gypsy Athila (471 hits 31.3%) Ty3-RT Ty3/gypsy Athila (329 hits 21.9%) Ty3-RH Ty3/gypsy Athila (174 hits 11.6%)	organelle/mitochondria (2 hits 0.133%)	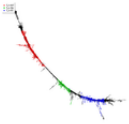	0.0939	0.532
49	CL49	223050	1487	0.212	15.60	Not available	Not available	LTR.Gypsy (**231hits, 9.46%**) Simple_repeat (105hits, 1.91%) Low_complexity (45hits, 1.04%)	LINE-ENDO NA NA (1 hits 0.0672%) Ty3-INT Ty3/gypsy Athila (1 hits 0.0672%)		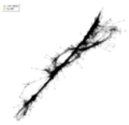	0.7380	7.196
50	CL50	220350	1469	0.210	15.80	Not available	Not available	Low_complexity (22hits, 0.379%) Simple_repeat (20hits, 0.344%)		organelle/plastid (1599 hits 109%) organelle/mitochondria (1 hits 0.0681%)	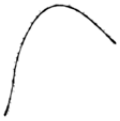	0.5189	2.042
51	CL51	218550	1457	0.208	16.00	Not available	Not available	Simple_repeat (**195hits, 4.87%**) LTR.Gypsy (23hits, 0.933%) LINE.L1 (20hits, 0.906%) Low_complexity (12hits, 0.31%)		organelle/plastid (1353 hits 92.9%) organelle/mitochondria (143 hits 9.81%)	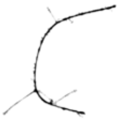	0.6990	2.128
52	CL52	215100	1434	0.205	16.20	Not available	Not available	LTR.Copia (**1128hits, 71.9%**) Simple_repeat (19hits, 0.328%) LTR.Gypsy (14hits, 0.182%) Low_complexity (7hits, 0.127%)	Ty1-INT Ty1/copia AleII (526 hits 36.7%) Ty1-GAG Ty1/copia AleII (119 hits 8.3%) Ty1-PROT Ty1/copia AleII (100 hits 6.97%) Ty1-PROT Ty1/copia Maximus/SIRE (9 hits 0.628%) Ty1-INT Ty1/copia AleI/Retrofit (1 hi......		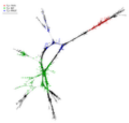	0.0943	0.558
							Not [illegible]	LTR.Gypsy (**623hits, 34.9%**) Simple_repeat (17hits, [illegible]	Ty3-GAG Ty3/gypsy Athila (209 hits 14.6%) [illegible]		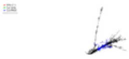		

53	CL53	214350	1429	0.204	16.40	Not available	Not<br>available	0.367%)<br>Low_complexity<br>(7hits, 0.167%)<br>LTR.Copia (3hits,<br>0.0569%)	Ty3-PROT Ty3/gypsy<br>Athila (149 hits 10.4%)<br>DTA-CD1 NA NA (1<br>hits 0.07%)		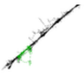	0.1036	0.490
54	CL54	213750	1425	0.204	16.60	Not available	Not<br>available	LTR.Copia (**96hits,<br>6.01%**)<br>LTR.Gypsy (35hits,<br>1.51%)<br>rRNA (12hits, 0.328%)<br>Simple_repeat (16hits,<br>0.282%)<br>Low_complexity<br>(1hits, 0.0145%)	Ty1-RT Ty1/copia Tork<br>(44 hits 3.09%)<br>Ty1-RH Ty1/copia Tork<br>(19 hits 1.33%)<br>Ty3-INT Ty3/gypsy<br>chromovirus (16 hits<br>1.12%)<br>Ty3-RT Ty3/gypsy<br>Ogre/Tat (5 hits 0.351%)<br>Ty1-RT Ty1/copia<br>Angela (1 hits 0.0702%)	organelle/mitochondria<br>(996 hits 69.9%)<br>organelle/plastid (8 hits<br>0.561%)	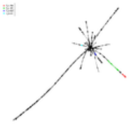	0.0770	0.421
55	CL55	208950	1393	0.199	16.80	Not available	Not<br>available	Simple_repeat (21hits,<br>0.682%)<br>LTR.Copia (8hits,<br>0.157%)		organelle/plastid (1341<br>hits 96.3%)<br>organelle/mitochondria<br>(22 hits 1.58%)	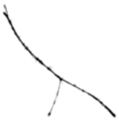	0.5524	2.584
56	CL56	208650	1391	0.199	17.00	Not available	Not<br>available	LTR.Gypsy (**212hits,<br>8.6%**)<br>LTR.Copia. (73hits,<br>1.83%)<br>Simple_repeat (33hits,<br>0.928%)<br>Low_complexity<br>(26hits, 0.785%)<br>LTR (13hits, 0.61%)<br>DNA.MULE.MuDR<br>(13hits, 0.215%)<br>LINE.L1 (1hits,<br>0.0273%)<br>......	Ty3-GAG Ty3/gypsy<br>Athila (86 hits 6.18%)		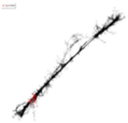	0.1283	1.078
57	CL57	207450	1383	0.198	17.20	Not available	Not<br>available	LTR.Copia (**247hits,<br>13.6%**)<br>rRNA (**199hits,<br>8.49%**)<br>Simple_repeat (3hits,<br>0.067%)		organelle/plastid (2770<br>hits 200%)<br>organelle/mitochondria<br>(2 hits 0.145%)	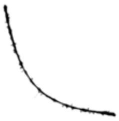	1.5600	4.772
58	CL58	205500	1370	0.196	17.40	Not available	Not<br>available	Simple_repeat<br>(155hits, 2.78%)<br>LTR.Copia (73hits,<br>1.8%)<br>Low_complexity<br>(4hits, 0.0618%)			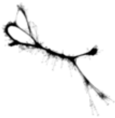	2.1300	7.372
59	CL59	203550	1357	0.194	17.60	Not available	Not<br>available	LTR.Copia (**100hits,<br>4.87%**)<br>Simple_repeat (31hits,<br>0.93%)<br>Low_complexity<br>(6hits, 0.167%)<br>LTR.Gypsy (1hits,<br>0.0197%)	Ty1-GAG Ty1/copia<br>Ivana/Oryco (1 hits<br>0.0737%)<br>Ty1-RT Ty1/copia AleII<br>(1 hits 0.0737%)	organelle/plastid (1366<br>hits 101%)<br>organelle/mitochondria<br>(17 hits 1.25%)	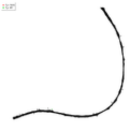	0.5066	2.137
60	CL60	197550	1317	0.188	17.80	Not available	Not<br>available	Simple_repeat<br>(**156hits, 4.49%**)<br>Low_complexity<br>(51hits, 1.4%)		organelle/plastid (1037<br>hits 78.7%)<br>organelle/mitochondria<br>(49 hits 3.72%)	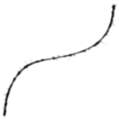	0.5939	1.822
61	CL61	194100	1294	0.185	18.00	Not available	Not<br>available	LTR.Copia (**334hits,<br>16.1%**)<br>Simple_repeat<br>(**258hits, 8.99%**)<br>LTR.Gypsy (8hits,<br>0.361%)	Ty1-RH Ty1/copia<br>Angela (29 hits 2.24%)<br>Ty1-RT Ty1/copia AleII<br>(2 hits 0.155%)<br>DTM-CD1 NA NA (1<br>hits 0.0773%)<br>Ty1-INT Ty1/copia<br>Angela (1 hits 0.0773%)<br>Ty1-INT Ty1/copia Tork<br>(1 hits 0.0773%)<br>Ty1-RT Ty1/co......		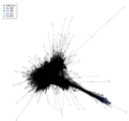	11.9200	37.560
62	CL62	193800	1292	0.185	18.20	Not available	Not<br>available	Simple_repeat<br>(116hits, 2.72%)	Ty1-RT Ty1/copia AleII<br>(1 hits 0.0774%)	organelle/plastid (2723<br>hits 211%)<br>organelle/mitochondria<br>(47 hits 3.64%)	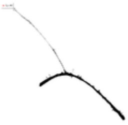	2.5190	6.579

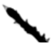

63	CL63	193350	1289	0.184	18.40	Not available	Not available	rRNA (**1288hits, 98.5%**)		45S_rDNA/25S_rDNA (1261 hits 97.8%)	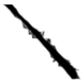	4.2860	11.790
64	CL64	193200	1288	0.184	18.50	Not available	Not available	Simple_repeat (123hits, 2.39%)<br>Low_complexity (44hits, 0.822%)		organelle/plastid (1097 hits 85.2%)<br>organelle/mitochondria (115 hits 8.93%)	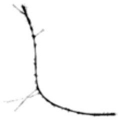	0.7449	2.484
65	CL65	188850	1259	0.180	18.70	Not available	Not available	DNA.MULE.MuDR (**73hits, 3.19%**)<br>LTR.Copia (14hits, 0.686%)<br>Low_complexity (17hits, 0.342%)<br>Simple_repeat (2hits, 0.0381%)		organelle/plastid (1215 hits 96.5%)<br>organelle/mitochondria (11 hits 0.874%)	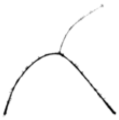	1.0380	3.018
66	CL66	185850	1239	0.177	18.90	Not available	Not available	LTR.Copia (47hits, 2.14%)<br>Simple_repeat (59hits, 1.22%)<br>Low_complexity (2hits, 0.127%)		organelle/plastid (1148 hits 92.7%)<br>organelle/mitochondria (53 hits 4.28%)	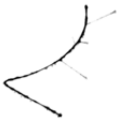	0.6555	3.632
67	CL67	185100	1234	0.176	19.10	Not available	Not available	Simple_repeat (27hits, 0.496%)		organelle/plastid (1417 hits 115%)	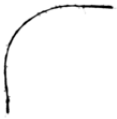	1.1850	3.566
68	CL68	183600	1224	0.175	19.30	Not available	Not available		LINE-RT NA NA (2 hits 0.163%)	organelle/plastid (2458 hits 201%)<br>organelle/mitochondria (1 hits 0.0817%)	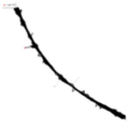	2.0660	7.516
69	CL69	181050	1207	0.172	19.40	Not available	Not available			organelle/plastid (2412 hits 200%)	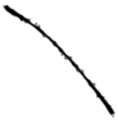	2.9340	7.374
70	CL70	180450	1203	0.172	19.60	Not available	Not available			organelle/plastid (1204 hits 100%)	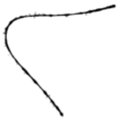	0.7950	2.743
71	CL71	177600	1184	0.169	19.80	Not available	Not available	LTR.Gypsy (**223hits, 12.2%**)<br>Simple_repeat (36hits, 1.03%)<br>Low_complexity (22hits, 0.681%)<br>LTR.Copia (2hits, 0.0586%)	Ty1-RT Ty1/copia Angela (1 hits 0.0845%)<br>Ty3-INT Ty3/gypsy Athila (1 hits 0.0845%)		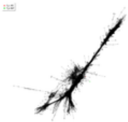	0.4563	3.378
72	CL72	175650	1171	0.167	19.90	Not available	Not available	LTR.Gypsy (**582hits, 44%**)<br>Low_complexity (53hits, 2.18%)<br>Simple_repeat (60hits, 2.05%)<br>LTR.Copia (3hits, 0.13%)	Ty3-INT Ty3/gypsy chromovirus (139 hits 11.9%)<br>Ty3-RT Ty3/gypsy chromovirus (123 hits 10.5%)<br>Ty3-GAG Ty3/gypsy chromovirus (117 hits 9.99%)<br>Ty3-RH Ty3/gypsy chromovirus (45 hits 3.84%)<br>Ty3-PROT Ty3/gypsy chro......	organelle/mitochondria (2 hits 0.171%)	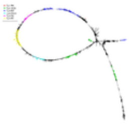	0.0176	0.085

73	CL73	174300 1162	0.166	20.10	Not available	Not available	RC.Helitron (**107hits, 6.98%**)		organelle/plastid (1187 hits 102%)	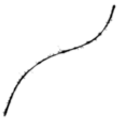	1.0260	3.442
74	CL74	172950 1153	0.165	20.30	Not available	Not available	Low_complexity (10hits, 0.317%) Simple_repeat (10hits, 0.304%)		organelle/plastid (1207 hits 105%)	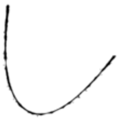	0.6096	2.082
75	CL75	172800 1152	0.165	20.40	Not available	Not available	Simple_repeat (100hits, 2.88%) Low_complexity (64hits, 2.24%) LTR.Gypsy (3hits, 0.111%)			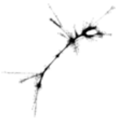	4.0000	16.320
76	CL76	172650 1151	0.164	20.60	Not available	Not available	Simple_repeat (46hits, 1.1%) Low_complexity (1hits, 0.0423%) tRNA (1hits, 0.0376%)		organelle/plastid (2298 hits 200%) organelle/mitochondria (3 hits 0.261%)	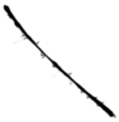	1.3610	5.560
77	CL77	170100 1134	0.162	20.80	Not available	Not available	LTR.Copia (5hits, 0.106%)		organelle/plastid (975 hits 86%) organelle/mitochondria (168 hits 14.8%)	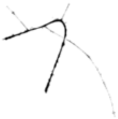	0.7505	3.175
78	CL78	169200 1128	0.161	20.90	Not available	Not available	LTR.Copia (**319hits, 18.3%**)		organelle/plastid (2269 hits 201%)	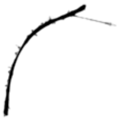	1.7400	4.965
79	CL79	166200 1108	0.158	21.10	Not available	Not available	DNA.hAT.Ac (**47hits, 3.55%**) Low_complexity (21hits, 0.481%) Simple_repeat (12hits, 0.288%)		organelle/plastid (1039 hits 93.8%) organelle/mitochondria (17 hits 1.53%)	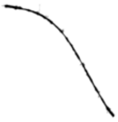	1.1150	3.610
80	CL80	166200 1108	0.158	21.20	Not available	Not available	Simple_repeat (2hits, 0.0403%)		organelle/plastid (2211 hits 200%) organelle/mitochondria (1 hits 0.0903%)	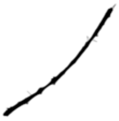	2.6390	7.852
81	CL81	164850 1099	0.157	21.40	Not available	Not available	LTR.Copia (**109hits, 7.69%**) DNA.CMC.EnSpm (21hits, 0.835%) LTR.Gypsy (4hits, 0.159%) Simple_repeat (2hits, 0.0388%)	Ty1-RT Ty1/copia unclass(AleI/Retrofit) (18 hits 1.64%) Ty1-RH Ty1/copia unclass(AleI/Retrofit) (13 hits 1.18%) Ty1-RT Ty1/copia AleII (13 hits 1.18%) Ty1-RH Ty1/copia Ivana/Oryco (12 hits 1.09%) Ty1-RT Ty1/c......	organelle/mitochondria (1114 hits 101%)	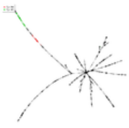	0.0209	0.091
82	CL82	162150 1081	0.154	21.50	Not available	Not available	LTR.Copia (**677hits, 58.5%**) Simple_repeat (9hits, 0.194%) Low_complexity (4hits, 0.17%) LTR.Gypsy (1hits, 0.0234%)	Ty1-RT Ty1/copia Angela (109 hits 10.1%) Ty1-INT Ty1/copia Angela (102 hits 9.44%) Ty1-RH Ty1/copia Angela (77 hits 7.12%) Ty1-PROT Ty1/copia Angela (39 hits 3.61%) Ty1-RT Ty1/copia Tork (11 hits 1.02%) Ty......		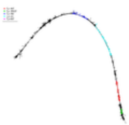	0.0326	0.278
83	CL83	160500 1070	0.153	21.70	Not available	Not available	LTR.Copia (**243hits, 18.1%**) Simple_repeat (**111hits, 3.2%**) Low_complexity (26hits, 0.629%) tRNA (1hits, 0.0349%)		organelle/plastid (1140 hits 107%)	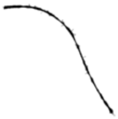	0.4642	1.776

84	CL84	158850	1059	0.151	21.90	Not available	Not available	LTR.Copia (3hits, 0.124%)		organelle/plastid (2363 hits 223%)	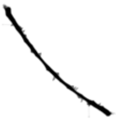	2.1890	7.932
85	CL85	156750	1045	0.149	22.00	Not available	Not available	Simple_repeat (43hits, 1%)<br>Low_complexity (15hits, 0.445%)		organelle/plastid (1048 hits 100%)<br>organelle/mitochondria (19 hits 1.82%)	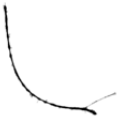	0.6549	2.679
86	CL86	156150	1041	0.149	22.10	Not available	Not available	Simple_repeat (31hits, 1.09%)		organelle/plastid (1016 hits 97.6%)	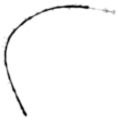	0.6719	1.921
87	CL87	156000	1040	0.149	22.30	Not available	Not available	LTR.Copia (**793hits, 70.5%**)<br>Simple_repeat (18hits, 0.432%)<br>Low_complexity (1hits, 0.0231%)	Ty1-RT Ty1/copia Angela (251 hits 24.1%)<br>Ty1-RH Ty1/copia Angela (108 hits 10.4%)<br>Ty1-INT Ty1/copia Angela (70 hits 6.73%)<br>Ty3-INT Ty3/gypsy chromovirus (9 hits 0.865%)<br>Ty1-RT Ty1/copia Tork (6 hits 0.577%)		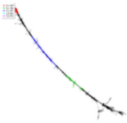	0.2604	1.346
88	CL88	149850	999	0.143	22.40	Not available	Not available	Simple_repeat (**126hits, 3.54%**)<br>Low_complexity (20hits, 0.543%)		organelle/plastid (1012 hits 101%)	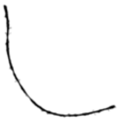	0.8456	2.703
89	CL89	149100	994	0.142	22.60	Not available	Not available	rRNA (**720hits, 67.1%**)<br>Simple_repeat (3hits, 0.0711%)	DTM-CD1 NA NA (48 hits 4.83%)	45S_rDNA/25S_rDNA (632 hits 63.6%)<br>45S_rDNA/5.8S_rDNA (131 hits 13.2%)	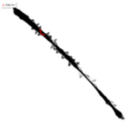	3.8020	7.847
90	CL90	148950	993	0.142	22.70	Not available	Not available	LTR.Gypsy (**328hits, 19.3%**)<br>Simple_repeat (**137hits, 4.51%**)<br>Low_complexity (71hits, 2.76%)<br>LTR.Copia (1hits, 0.0369%)			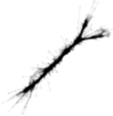	0.1556	2.316
91	CL91	146250	975	0.139	22.90	Not available	Not available	Simple_repeat (72hits, 1.96%)<br>RC.Helitron (41hits, 1.8%)<br>Low_complexity (1hits, 0.0403%)<br>LTR.Copia (1hits, 0.0274%)		organelle/plastid (895 hits 91.8%)<br>organelle/mitochondria (14 hits 1.44%)	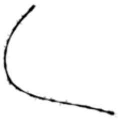	0.6367	1.949
92	CL92	145500	970	0.139	23.00	Not available	Not available	rRNA (**153hits, 9.44%**)	Ty1-INT Ty1/copia AleI/Retrofit (1 hits 0.103%)	organelle/plastid (1936 hits 200%)<br>organelle/mitochondria (5 hits 0.515%)	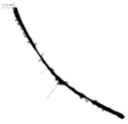	2.2630	6.598
93	CL93	144300	962	0.137	23.10	Not available	Not available	LTR.Copia (**874hits, 84.3%**)<br>Simple_repeat (11hits, 0.272%)<br>Low_complexity (1hits, 0.0347%)<br>LTR.Gypsy (1hits, 0.0173%)	Ty1-RT Ty1/copia AleII (350 hits 36.4%)<br>Ty1-RH Ty1/copia AleII (204 hits 21.2%)<br>Ty1-RT Ty1/copia Ivana/Oryco (7 hits 0.728%)<br>Ty1-RT Ty1/copia AleI/Retrofit (2 hits 0.208%)<br>Ty1-RH Ty1/copia	organelle/mitochondria (21 hits 2.18%)	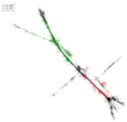	0.2842	0.936

								[illegible]<br>Maximus/SIRE (1 hit......				
94	CL94	142800 952	0.136	23.30	Not available	Not available	Low_complexity (38hits, 1.09%)<br>Simple_repeat (32hits, 0.917%)		organelle/plastid (1000 hits 105%)	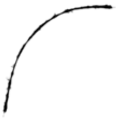	0.7796	4.097
95	CL95	142500 950	0.136	23.40	Not available	Not available	Simple_repeat (41hits, 1.61%)			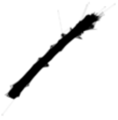	1.2260	11.260
96	CL96	138300 922	0.132	23.50	Not available	Not available			organelle/plastid (916 hits 99.3%)	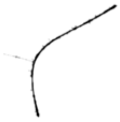	1.2670	3.796
97	CL97	136050 907	0.130	23.70	Not available	Not available	LTR.Gypsy (**487hits, 48.6%**)<br>Simple_repeat (33hits, 0.915%)<br>Low_complexity (4hits, 0.143%)<br>LTR.Copia (2hits, 0.0897%)<br>LTR (1hits, 0.0353%)<br>LTR.Cassandra (1hits, 0.0294%)	Ty3-RT Ty3/gypsy chromovirus (176 hits 19.4%)<br>Ty3-RH Ty3/gypsy chromovirus (105 hits 11.6%)<br>Ty3-PROT Ty3/gypsy chromovirus (18 hits 1.98%)<br>Ty3-INT Ty3/gypsy chromovirus (12 hits 1.32%)<br>LINE-RT NA NA (1 hits 0.11%)		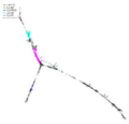	0.0255	0.110
98	CL98	134700 898	0.128	23.80	Not available	Not available	LTR.Copia (**637hits, 63.1%**)<br>LTR.Gypsy (19hits, 1.49%)<br>DNA.TcMar.Tc1 (2hits, 0.172%)<br>LTR.Caulimovirus (1hits, 0.0468%)<br>Simple_repeat (2hits, 0.0319%)	Ty1-RT Ty1/copia unclass(AleI/Retrofit) (105 hits 11.7%)<br>Ty1-INT Ty1/copia unclass(AleI/Retrofit) (100 hits 11.1%)<br>Ty1-RH Ty1/copia unclass(AleI/Retrofit) (62 hits 6.9%)<br>Ty1-PROT Ty1/copia unclass(AleI/Retrofit)......	organelle/mitochondria (26 hits 2.9%)	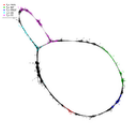	0.0000	0.000
99	CL99	130350 869	0.124	23.90	Not available	Not available	Simple_repeat (52hits, 2.14%)<br>Low_complexity (5hits, 0.183%)		organelle/plastid (874 hits 101%)	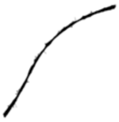	1.4970	4.833
100	CL100	129150 861	0.123	24.00	Not available	Not available	Simple_repeat (**87hits, 5.03%**)<br>Low_complexity (16hits, 0.746%)		organelle/plastid (704 hits 81.8%)	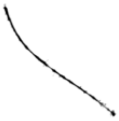	0.5619	3.368
101	CL101	128700 858	0.123	24.20	Not available	Not available	Simple_repeat (46hits, 1.46%)<br>Low_complexity (30hits, 0.932%)		organelle/plastid (717 hits 83.6%)<br>organelle/mitochondria (175 hits 20.4%)	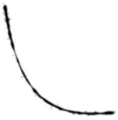	0.5202	3.030
102	CL102	123900 826	0.118	24.30	Not available	Not available	LTR.Copia (**725hits, 80.3%**)<br>Simple_repeat (1hits, 0.0274%)	Ty1-RT Ty1/copia Tork (178 hits 21.5%)<br>Ty1-INT Ty1/copia Tork (162 hits 19.6%)<br>Ty1-RH Ty1/copia Tork (95 hits 11.5%)<br>Ty1-PROT Ty1/copia Tork (41 hits 4.96%)<br>Ty1-RT Ty1/copia AleII (8 hits 0.969%)<br>Ty1-PROT		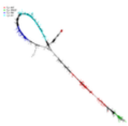	0.0965	0.605
										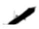		

103	CL103	122250	815	0.116	24.40	Not available	Not available	Simple_repeat (90hits, 1.83%) LTR.Copia (1hits, 0.0278%)		organelle/plastid (1732 hits 213%)	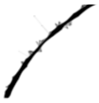	2.5740	8.834
104	CL104	121650	811	0.116	24.50	Not available	Not available	Simple_repeat (22hits, 0.734%) Low_complexity (5hits, 0.225%)		organelle/plastid (771 hits 95.1%) organelle/mitochondria (39 hits 4.81%)	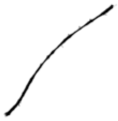	1.1430	4.192
105	CL105	120600	804	0.115	24.60	Not available	Not available	LTR.Gypsy (**480hits, 49.9%**) LTR.Copia. (19hits, 0.915%)	Ty3-PROT Ty3/gypsy Athila (118 hits 14.7%) Ty3-RT Ty3/gypsy Athila (40 hits 4.98%) Ty3-INT Ty3/gypsy Athila (37 hits 4.6%)		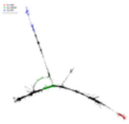	0.2294	1.493
106	CL106	118500	790	0.113	24.70	Not available	Not available	Simple_repeat (10hits, 0.603%)		organelle/plastid (786 hits 99.5%) organelle/mitochondria (42 hits 5.32%)	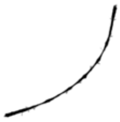	0.6284	2.532
107	CL107	117450	783	0.112	24.90	Not available	Not available	Simple_repeat (62hits, 2.25%) Low_complexity (1hits, 0.046%)	LINE-RT NA NA (26 hits 3.32%)	organelle/plastid (803 hits 103%)	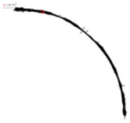	1.1770	5.364
108	CL108	117450	783	0.112	25.00	Not available	Not available	LTR.Copia (**689hits, 83.2%**) Simple_repeat (7hits, 0.279%)	Ty1-RT Ty1/copia Angela (125 hits 16%) Ty1-INT Ty1/copia Angela (74 hits 9.45%) Ty1-RH Ty1/copia Angela (64 hits 8.17%) Ty1-GAG Ty1/copia Angela (45 hits 5.75%) Ty1-PROT Ty1/copia Angela (30 hits 3.83%) Ty......		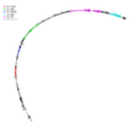	0.0216	0.128
109	CL109	111300	742	0.106	25.10	Not available	Not available	Simple_repeat (1hits, 0.0422%)		organelle/plastid (741 hits 99.9%)	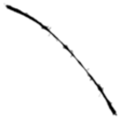	1.2700	4.178
110	CL110	109650	731	0.104	25.20	Not available	Not available	Simple_repeat (**46hits, 3.05%**)		organelle/plastid (668 hits 91.4%)	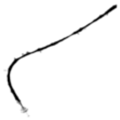	0.6818	2.326
111	CL111	109050	727	0.104	25.30	Not available	Not available	Simple_repeat (**85hits, 3.29%**) Low_complexity (3hits, 0.157%)		organelle/plastid (709 hits 97.5%)	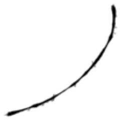	0.9741	5.227
112	CL112	107700	718	0.103	25.40	Not available	Not	LTR.Copia (**588hits, 76.4%**) Simple_repeat (31hits,	Ty1-INT Ty1/copia Angela (131 hits 18.2%) Ty1-GAG Ty1/copia Angela (92 hits 12.8%) Ty1-PROT Ty1/copia Angela (33 hits 4.6%)			0.4244	1.532

							available	1.35%) Low_complexity (4hits, 0.214%)	Ty1-PROT Ty1/copia TAR (30 hits 4.18%) Ty1-INT Ty1/copia AleII (5 hits 0.696%) Ty......		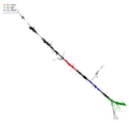		
113	CL113	106500	710	0.101	25.50	Not available	Not available	LTR.Copia (**548hits, 68.8%**) Low_complexity (1hits, 0.0479%)	Ty1-INT Ty1/copia AleII (216 hits 30.4%) Ty1-GAG Ty1/copia AleII (108 hits 15.2%) Ty1-PROT Ty1/copia AleII (55 hits 7.75%) Ty1-PROT Ty1/copia Ivana/Oryco (1 hits 0.141%) Ty1-PROT Ty1/copia Maximus/SIRE (1 hit......	organelle/plastid (1 hits 0.141%)	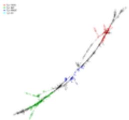	0.1642	0.563
114	CL114	99300	662	0.095	25.60	Not available	Not available	Simple_repeat (66hits, 2.79%) Low_complexity (5hits, 0.254%)		organelle/plastid (590 hits 89.1%) organelle/mitochondria (65 hits 9.82%)	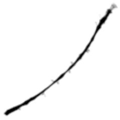	0.5980	4.532
115	CL115	98850	659	0.094	25.70	Not available	Not available	LTR.Gypsy (**203hits, 13.3%**) RC.Helitron (1hits, 0.0607%) Simple_repeat (1hits, 0.0486%)			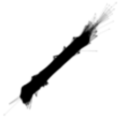	1.9700	12.140
116	CL116	98850	659	0.094	25.80	Not available	Not available	Simple_repeat (49hits, 1.87%) LTR.Copia (12hits, 0.909%) DNA.hAT.Tag1 (2hits, 0.18%) LTR.Gypsy (1hits, 0.0931%) Low_complexity (1hits, 0.0395%)			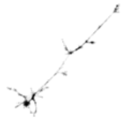	0.0000	0.000
117	CL117	97500	650	0.093	25.90	Not available	Not available	LTR.Copia (**373hits, 49%**) LTR.Gypsy (**101hits, 11.3%**) Simple_repeat (43hits, 1.37%) LTR.Caulimovirus (3hits, 0.211%) DNA.TcMar.Tc1 (1hits, 0.103%) Low_complexity (1hits, 0.0656%)	Ty1-INT Ty1/copia unclass(AleI/Retrofit) (99 hits 15.2%) Ty1-PROT Ty1/copia unclass(AleI/Retrofit) (26 hits 4%) Ty1-RT Ty1/copia Ivana/Oryco (19 hits 2.92%) Ty1-GAG Ty1/copia AleI/Retrofit (17 hits 2.62%) Ty1......	organelle/mitochondria (1 hits 0.154%)	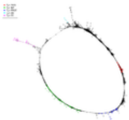	0.0000	0.000
118	CL118	97200	648	0.093	26.00	Not available	Not available	RC.Helitron (29hits, 2.81%)	DTC-CD1 NA NA (1 hits 0.154%)	organelle/plastid (626 hits 96.6%) organelle/mitochondria (9 hits 1.39%)	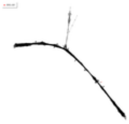	1.4300	5.401
119	CL119	96000	640	0.091	26.10	Not available	Not available	DNA.MULE.MuDR (**115hits, 14.7%**) Low_complexity (2hits, 0.0938%) Simple_repeat (1hits, 0.0323%)	DTM-CD1 NA NA (196 hits 30.6%) DTC-CD1 NA NA (2 hits 0.312%)		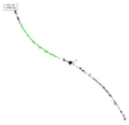	0.0000	0.000
120	CL120	95700	638	0.091	26.10	Not available	Not available	DNA.PIF.Harbinger (20hits, 1.89%) Simple_repeat (28hits, 1.18%) Low_complexity (25hits, 1.14%) LTR.Copia (2hits, 0.132%)	Ty1-INT Ty1/copia AleII (1 hits 0.157%)		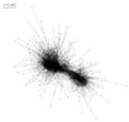	0.0000	0.000
121	CL121	94650	631	0.090	26.20	Not available	Not available	LTR.Copia (14hits, 1.06%) LTR.Gypsy (11hits, 0.827%) Simple_repeat (18hits, 0.558%) Low_complexity (4hits, 0.278%)	Ty1-RH Ty1/copia AleII (3 hits 0.475%)	organelle/mitochondria (492 hits 78%)	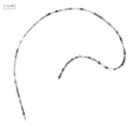	0.0000	0.000

122	CL122	93750	625	0.089	26.30	Not available	Not available	LTR.Gypsy (**147hits, 8.45%**)<br>Simple_repeat (29hits, 1.42%)<br>Low_complexity (9hits, 0.458%)<br>LTR.Copia (2hits, 0.109%)<br>rRNA (1hits, 0.0309%)			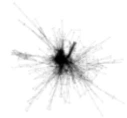	2.4430	7.520
123	CL123	88500	590	0.084	26.40	Not available	Not available	LTR.Gypsy (15hits, 1.03%)<br>Simple_repeat (14hits, 0.493%)<br>Low_complexity (2hits, 0.0701%)<br>LTR.Copia (1hits, 0.0475%)	Ty1-RT Ty1/copia Angela (1 hits 0.169%)		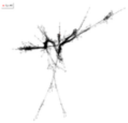	2.0750	6.949
124	CL124	86850	579	0.083	26.50	Not available	Not available	LTR.Copia (4hits, 0.216%)<br>Simple_repeat (2hits, 0.061%)<br>LTR.Gypsy (1hits, 0.0391%)	Ty1-INT Ty1/copia AleII (1 hits 0.173%)		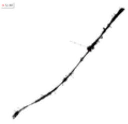	2.1820	5.181
125	CL125	84150	561	0.080	26.60	Not available	Not available	LTR.Copia (**362hits, 54.1%**)<br>Simple_repeat (2hits, 0.0428%)<br>DNA.hAT.Tag1 (1hits, 0.0368%)	Ty1-INT Ty1/copia Angela (121 hits 21.6%)<br>Ty1-RT Ty1/copia Angela (40 hits 7.13%)<br>Ty1-INT Ty1/copia AleII (10 hits 1.78%)<br>Ty1-INT Ty1/copia Ivana/Oryco (2 hits 0.357%)<br>Ty1-RT Ty1/copia Ivana/Oryco (1 hits 0.178%)		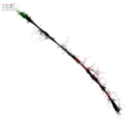	2.0040	6.952
126	CL126	83850	559	0.080	26.60	Not available	Not available	Simple_repeat (49hits, 1.7%)<br>Low_complexity (7hits, 0.329%)<br>LTR.Copia (1hits, 0.0489%)			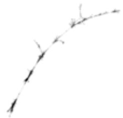	0.0000	0.000
127	CL127	82350	549	0.078	26.70	Not available	Not available	LTR.Gypsy (21hits, 2.75%)<br>Simple_repeat (5hits, 0.225%)<br>Low_complexity (2hits, 0.0959%)	Ty3-INT Ty3/gypsy chromovirus (34 hits 6.19%)		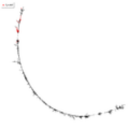	0.0000	0.000
128	CL128	76650	511	0.073	26.80	Not available	Not available	Simple_repeat (39hits, 1.66%)<br>LTR (3hits, 0.364%)<br>Low_complexity (4hits, 0.307%)<br>LTR.Copia (4hits, 0.223%)<br>LTR.Gypsy (2hits, 0.181%)			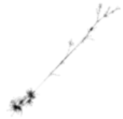	0.0000	0.000
129	CL129	75450	503	0.072	26.90	Not available	Not available	Simple_repeat (41hits, 2.74%)<br>Low_complexity (24hits, 1.6%)<br>LTR.Gypsy (11hits, 1.08%)<br>LTR.Copia (1hits, 0.0782%)<br>DNA.MULE.MuDR (1hits, 0.0557%)	DTM-CD1 NA NA (1 hits 0.199%)		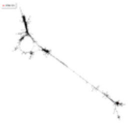	1.1360	2.982
130	CL130	74250	495	0.071	26.90	Not available	Not available	Simple_repeat (**500hits, 92.2%**)<br>Low_complexity (9hits, 0.514%)<br>LINE.L1 (1hits, 0.191%)		organelle/plastid (10 hits 2.02%)	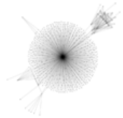	0.0000	0.000
131	CL131	73650	491	0.070	27.00	Not available	Not available	LTR.Gypsy (**156hits, 22.9%**)<br>LTR.Copia (4hits, 0.266%)	Ty3-GAG Ty3/gypsy Athila (159 hits 32.4%)<br>Ty1-GAG Ty1/copia AleII (1 hits 0.204%)		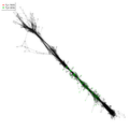	1.4090	4.277

132	CL132	71250	475	0.068	27.10	Not available	Not available	Simple_repeat (**80hits, 5.59%**)<br>Low_complexity (**44hits, 4.16%**)<br>LTR.Gypsy (1hits, 0.0407%)			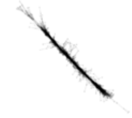	0.6136	3.368
133	CL133	70200	468	0.067	27.10	Not available	Not available	LTR.Gypsy (**101hits, 19.8%**)<br>rRNA (**35hits, 3.97%**)<br>LTR.Copia (13hits, 2.36%)<br>DNA.TcMar.Stowaway (14hits, 1.34%)<br>DNA.CMC.EnSpm (12hits, 0.775%)<br>Low_complexity (3hits, 0.175%)	Ty3-INT Ty3/gypsy chromovirus (61 hits 13%)<br>Ty3-CHDII Ty3/gypsy chromovirus (12 hits 2.56%)	organelle/mitochondria (457 hits 97.6%)	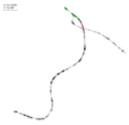	0.0437	0.214
134	CL134	69150	461	0.066	27.20	Not available	Not available	LTR.Gypsy (19hits, 2.73%)	Ty3-GAG Ty3/gypsy Ogre/Tat (15 hits 3.25%)	organelle/mitochondria (149 hits 32.3%)	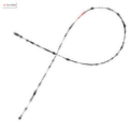	0.0000	0.000
135	CL135	68550	457	0.065	27.30	Not available	Not available	LTR.Copia (**386hits, 66.3%**)<br>LTR (**43hits, 3.51%**)<br>LTR.Gypsy (4hits, 0.443%)<br>LTR.Cassandra (5hits, 0.324%)<br>Simple_repeat (2hits, 0.0992%)	Ty1-RH Ty1/copia Tork (49 hits 10.7%)<br>Ty1-RT Ty1/copia Tork (23 hits 5.03%)<br>Ty1-GAG Ty1/copia Tork (16 hits 3.5%)<br>Ty1-PROT Ty1/copia AleI/Retrofit (1 hits 0.219%)	organelle/mitochondria (16 hits 3.5%)	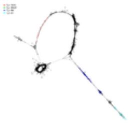	0.0000	0.000
136	CL136	67350	449	0.064	27.30	Not available	Not available	Simple_repeat (4hits, 0.226%)<br>RC.Helitron (2hits, 0.137%)		organelle/mitochondria (353 hits 78.6%)	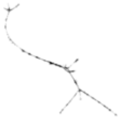	0.2198	1.336
137	CL137	65850	439	0.063	27.40	Not available	Not available	DNA.MULE.MuDR (**164hits, 29.8%**)<br>Simple_repeat (4hits, 0.199%)<br>Low_complexity (1hits, 0.0683%)	DTM-CD1 NA NA (218 hits 49.7%)		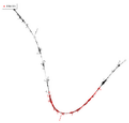	0.0000	0.000
138	CL138	64950	433	0.062	27.50	Not available	Not available	LINE.L1 (**200hits, 39.2%**)<br>Simple_repeat (5hits, 0.209%)<br>LTR.Gypsy (1hits, 0.125%)<br>Low_complexity (1hits, 0.0693%)	LINE-RT NA NA (107 hits 24.7%)		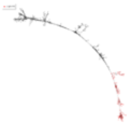	0.0000	0.000
139	CL139	62250	415	0.059	27.50	Not available	Not available	Simple_repeat (**54hits, 5.35%**)<br>Low_complexity (19hits, 2.23%)			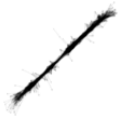	0.3570	3.373
140	CL140	60300	402	0.057	27.60	Not available	Not available	LTR.Copia (**216hits, 50.8%**)	Ty1-RT Ty1/copia Tork (114 hits 28.4%)<br>Ty1-INT Ty1/copia Tork (34 hits 8.46%)<br>Ty1-RH Ty1/copia Tork (28 hits 6.97%)	organelle/mitochondria (51 hits 12.7%)	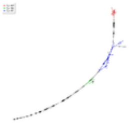	0.0000	0.000
141	CL141	57150	381	0.054	27.60	Not available	Not available	LTR.Copia (6hits, 0.49%)<br>Simple_repeat (1hits, 0.098%)		organelle/mitochondria (153 hits 40.2%)<br>organelle/plastid (64 hits 16.8%)	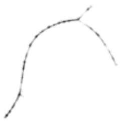	0.0000	0.000
142	CL142	56100	374	0.053	27.70	Not available	Not available	Simple_repeat (12hits, 0.524%)		organelle/mitochondria (416 hits 111%)	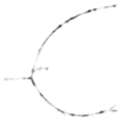	0.0000	0.000

143	CL143	56100	374	0.053	27.70	Not available	Not available	LTR.Copia (9hits, 0.544%)		organelle/mitochondria (160 hits 42.8%)	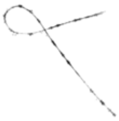	0.0425	0.267
144	CL144	55350	369	0.053	27.80	Not available	Not available	Simple_repeat (9hits, 0.58%) Low_complexity (1hits, 0.0488%)			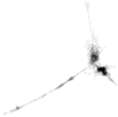	0.0000	0.000
145	CL145	52800	352	0.050	27.80	Not available	Not available	Low_complexity (1hits, 0.0625%)	Ty3-GAG Ty3/gypsy Ogre/Tat (3 hits 0.852%) DTA-CD1 NA NA (1 hits 0.284%)	organelle/mitochondria (266 hits 75.6%)	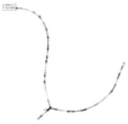	0.0000	0.000
146	CL146	51150	341	0.049	27.90	Not available	Not available	LTR.Gypsy (**59hits, 13.1%**) Simple_repeat (9hits, 0.739%) Low_complexity (5hits, 0.514%)			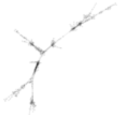	0.0000	0.000
147	CL147	50100	334	0.048	27.90	Not available	Not available	DNA.MULE.MuDR (11hits, 1.36%) Simple_repeat (13hits, 1.12%) Low_complexity (6hits, 0.469%)			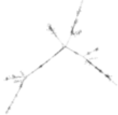	0.0000	0.000
148	CL148	49800	332	0.047	28.00	Not available	Not available	LTR.Gypsy (**160hits, 45.1%**) Simple_repeat (12hits, 0.773%)	Ty3-RH Ty3/gypsy chromovirus (39 hits 11.7%) Ty3-RT Ty3/gypsy chromovirus (37 hits 11.1%) Ty3-INT Ty3/gypsy chromovirus (35 hits 10.5%)	organelle/mitochondria (15 hits 4.52%)	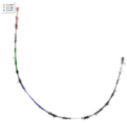	0.0961	0.602
149	CL149	47250	315	0.045	28.00	Not available	Not available	Simple_repeat (9hits, 0.586%) Low_complexity (4hits, 0.337%) DNA.MULE.MuDR (1hits, 0.135%)			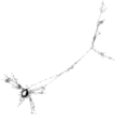	0.0000	0.000
150	CL150	45150	301	0.043	28.10	Not available	Not available	LTR.Copia (**240hits, 71.7%**) Simple_repeat (7hits, 0.525%) Low_complexity (1hits, 0.0753%)	Ty1-INT Ty1/copia TAR (63 hits 20.9%) Ty1-GAG Ty1/copia TAR (19 hits 6.31%) Ty1-INT Ty1/copia Angela (4 hits 1.33%) Ty1-PROT Ty1/copia AleII (4 hits 1.33%) Ty1-PROT Ty1/copia TAR (3 hits 0.997%) Ty1-INT Ty......		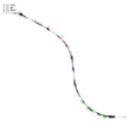	0.0000	0.000
151	CL151	45000	300	0.043	28.10	Not available	Not available	Simple_repeat (**25hits, 3.71%**) Low_complexity (10hits, 1.07%)		organelle/plastid (301 hits 100%)	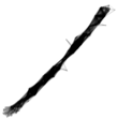	1.6360	11.000
152	CL152	44400	296	0.042	28.20	Not available	Not available	Low_complexity (3hits, 0.338%) Simple_repeat (3hits, 0.282%)			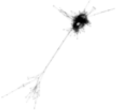	0.0000	0.000
153	CL153	43350	289	0.041	28.20	Not available	Not available	Satellite (**82hits, 15.8%**) Simple_repeat (1hits, 0.0554%)		5S_rDNA/5S_rDNA (83 hits 28.7%)	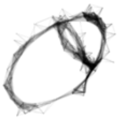	0.0000	0.000

154	CL154	43200	288	0.041	28.20	Not available	Not available	Simple_repeat (**81hits, 18.2%**)<br>Low_complexity (6hits, 0.699%)		organelle/plastid (10 hits 3.47%)	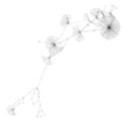	0.2681	0.347
155	CL155	41700	278	0.040	28.30	Not available	Not available	LTR.Copia (**222hits, 75%**)<br>Low_complexity (5hits, 0.513%)<br>Simple_repeat (2hits, 0.223%)	Ty1-RT Ty1/copia TAR (67 hits 24.1%)<br>Ty1-RH Ty1/copia TAR (31 hits 11.2%)<br>Ty1-RT Ty1/copia Ivana/Oryco (3 hits 1.08%)<br>Ty1-RT Ty1/copia AleI/Retrofit (2 hits 0.719%)<br>Ty1-RT Ty1/copia AleII (2 hits 0.719%)		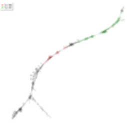	0.2035	0.719
156	CL156	39000	260	0.037	28.30	Not available	Not available	DNA.hAT.Ac (**106hits, 33.7%**)<br>DNA.PIF.Harbinger (1hits, 0.221%)<br>Simple_repeat (2hits, 0.105%)	DTA-CD1 NA NA (88 hits 33.8%)	organelle/mitochondria (1 hits 0.385%)	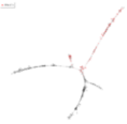	0.0000	0.000
157	CL157	37650	251	0.036	28.40	Not available	Not available			organelle/mitochondria (293 hits 117%)	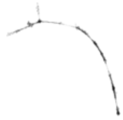	0.0596	0.398
158	CL158	37050	247	0.035	28.40	Not available	Not available	LTR.Gypsy (**217hits, 81.6%**)<br>Simple_repeat (4hits, 0.494%)<br>Low_complexity (1hits, 0.154%)	Ty3-INT Ty3/gypsy chromovirus (145 hits 58.7%)<br>Ty3-CHDII Ty3/gypsy chromovirus (19 hits 7.69%)<br>Ty3-RH Ty3/gypsy chromovirus (2 hits 0.81%)		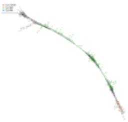	0.0000	0.000
159	CL159	36600	244	0.035	28.40	Not available	Not available	LTR.Copia (**86hits, 27.9%**)<br>DNA.CMC.EnSpm (9hits, 0.959%)	Ty1-GAG Ty1/copia AleII (33 hits 13.5%)<br>Ty1-RH Ty1/copia AleII (27 hits 11.1%)	organelle/mitochondria (176 hits 72.1%)	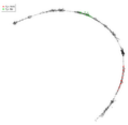	0.0000	0.000
160	CL160	34500	230	0.033	28.50	Not available	Not available	LTR.Gypsy (**19hits, 6.25%**)	Ty3-GAG Ty3/gypsy chromovirus (16 hits 6.96%)<br>Ty3-PROT Ty3/gypsy Ogre/Tat (5 hits 2.17%)	organelle/mitochondria (78 hits 33.9%)	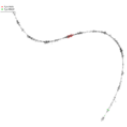	0.0000	0.000
161	CL161	34050	227	0.032	28.50	Not available	Not available	LTR.Gypsy (**38hits, 12.4%**)<br>Low_complexity (1hits, 0.106%)			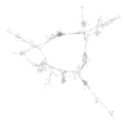	1.0930	1.762
162	CL162	34050	227	0.032	28.50	Not available	Not available	Simple_repeat (3hits, 0.256%)		organelle/mitochondria (81 hits 35.7%)	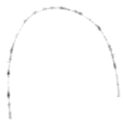	0.1168	0.441
163	CL163	33750	225	0.032	28.60	Not available	Not available	LTR.Copia (**220hits, 91.2%**)<br>DNA.hAT.Tip100 (1hits, 0.21%)	Ty1-INT Ty1/copia TAR (91 hits 40.4%)<br>Ty1-PROT Ty1/copia TAR (31 hits 13.8%)<br>Ty1-PROT Ty1/copia AleII (4 hits 1.78%)<br>Ty1-PROT Ty1/copia Tork (2 hits 0.889%)<br>Ty1-INT Ty1/copia Angela (1 hits 0.444%)		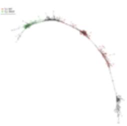	0.3018	1.333
								Simple_repeat (12hits, 2.71%)<br>[illegible]					

164	CL164	33450	223	0.032	28.60	Not available	Not available	Low_complexity (3hits, 0.601%) DNA.MULE.MuDR (1hits, 0.389%) LTR (1hits, 0.152%) LTR.Copia (1hits, 0.123%)	Ty3-GAG Ty3/gypsy chromovirus (1 hits 0.448%)		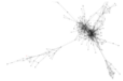	0.0000	0.000
165	CL165	32700	218	0.031	28.60	Not available	Not available	LTR.Gypsy (**12hits, 4.66%**) Simple_repeat (1hits, 0.116%)	Ty3-RT Ty3/gypsy Ogre/Tat (9 hits 4.13%)	organelle/mitochondria (31 hits 14.2%)	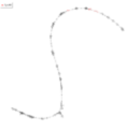	0.0000	0.000
166	CL166	32550	217	0.031	28.70	Not available	Not available	LTR.Gypsy (**35hits, 8.17%**) LTR.Copia. (13hits, 2.25%) LTR (1hits, 0.298%) Low_complexity (1hits, 0.144%)			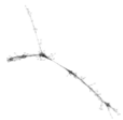	0.9852	2.304
167	CL167	31500	210	0.030	28.70	Not available	Not available	LTR.Gypsy (**45hits, 16.5%**) Simple_repeat (5hits, 0.578%) Low_complexity (1hits, 0.124%)			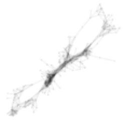	0.0000	0.000
168	CL168	31050	207	0.030	28.70	Not available	Not available	Simple_repeat (9hits, 1.83%) Low_complexity (1hits, 0.129%)			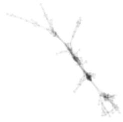	0.0000	0.000
169	CL169	30900	206	0.029	28.70	Not available	Not available	LTR.Copia (**178hits, 78.1%**) Simple_repeat (4hits, 0.505%)	Ty1-INT Ty1/copia Bianca (103 hits 50%) Ty1-PROT Ty1/copia Bianca (23 hits 11.2%)		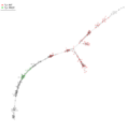	0.0000	0.000
170	CL170	29700	198	0.028	28.80	Not available	Not available	Simple_repeat (4hits, 0.455%)		organelle/mitochondria (218 hits 110%)	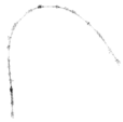	0.0000	0.000
171	CL171	29250	195	0.028	28.80	Not available	Not available	LINE.L1 (**68hits, 27.9%**) Low_complexity (1hits, 0.154%) Simple_repeat (2hits, 0.13%)			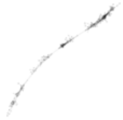	0.0000	0.000
172	CL172	29100	194	0.028	28.80	Not available	Not available	Simple_repeat (1hits, 0.0928%)		organelle/mitochondria (217 hits 112%)	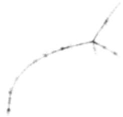	0.0000	0.000
173	CL173	28650	191	0.027	28.90	Not available	Not available	LTR.Copia (**193hits, 95.8%**)	Ty1-RT Ty1/copia Ivana/Oryco (93 hits 48.7%) Ty1-RH Ty1/copia Ivana/Oryco (62 hits 32.5%)	organelle/mitochondria (23 hits 12%)	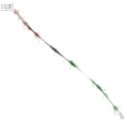	0.0000	0.000
174	CL174	28200	188	0.027	28.90	Not available	Not available	LTR.Copia (3hits, 0.649%)		organelle/mitochondria (115 hits 61.2%)	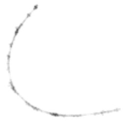	0.0000	0.000
175	CL175	27900	186	0.027	28.90	Not available	Not available	Simple_repeat (4hits, 0.717%) Low_complexity (2hits, 0.28%)		organelle/mitochondria (104 hits 55.9%)	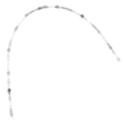	0.0000	0.000
								LTR.Copia (**47hits** [illegible]					

176	CL176	26850	179	0.026	28.90	Not available	Not available	LTR.Copia (**47hits, 20.9%**)<br>LTR.Gypsy (2hits, 0.484%)<br>Low_complexity (1hits, 0.153%)<br>Simple_repeat (1hits, 0.13%)	Ty1-INT Ty1/copia AleII (1 hits 0.559%)		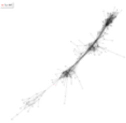	0.1015	0.559
177	CL177	26700	178	0.025	29.00	Not available	Not available	LTR.Copia (**138hits, 72.6%**)	Ty1-INT Ty1/copia Tork (56 hits 31.5%)<br>Ty1-PROT Ty1/copia Tork (49 hits 27.5%)<br>Ty1-GAG Ty1/copia Tork (5 hits 2.81%)	organelle/mitochondria (17 hits 9.55%)	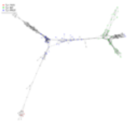	0.0000	0.000
178	CL178	26400	176	0.025	29.00	Not available	Not available	Simple_repeat (4hits, 0.78%)			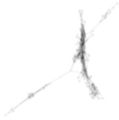	0.0000	0.000
179	CL179	25800	172	0.025	29.00	Not available	Not available	LTR.Copia (**15hits, 5.4%**)		organelle/mitochondria (6 hits 3.49%)	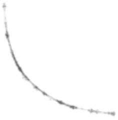	0.0000	0.000
180	CL180	25500	170	0.024	29.00	Not available	Not available					4.7420	14.710
181	CL181	25350	169	0.024	29.10	Not available	Not available	Simple_repeat (**41hits, 10.7%**)<br>DNA.hAT.Ac (**36hits, 10.4%**)<br>Low_complexity (**23hits, 5.7%**)<br>LTR.Copia (**11hits, 3.55%**)			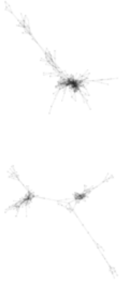	0.0000	0.000
182	CL182	25050	167	0.024	29.10	Not available	Not available	Low_complexity (6hits, 0.766%)<br>Simple_repeat (1hits, 0.0758%)		organelle/mitochondria (100 hits 59.9%)	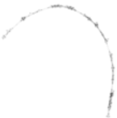	0.0000	0.000
183	CL183	24900	166	0.024	29.10	Not available	Not available	Simple_repeat (1hits, 0.145%)		organelle/mitochondria (179 hits 108%)	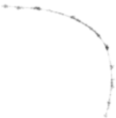	0.0000	0.000
184	CL184	24750	165	0.024	29.10	Not available	Not available	LTR.Copia (**146hits, 71.8%**)	Ty1-GAG Ty1/copia Tork (28 hits 17%)<br>Ty1-RH Ty1/copia AleII (1 hits 0.606%)		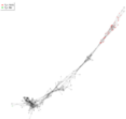	0.0000	0.000
185	CL185	24150	161	0.023	29.20	Not available	Not available	LTR.Copia (**123hits, 64.4%**)<br>Simple_repeat (3hits, 0.211%)	Ty1-INT Ty1/copia Ivana/Oryco (64 hits 39.8%)<br>Ty1-PROT Ty1/copia Ivana/Oryco (21 hits 13%)		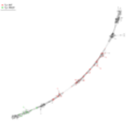	0.0000	0.000
186	CL186	24000	160	0.023	29.20	Not available	Not available	DNA.CMC.EnSpm (**74hits, 39.9%**)<br>Simple_repeat (7hits, 1.25%)			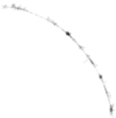	0.0000	0.000

187	CL187	23550	157	0.022	29.20	Not available	Not available	LTR.Copia (**48hits, 25.3%**)	Ty1-GAG Ty1/copia AleII (65 hits 41.4%)		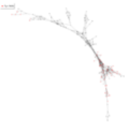	0.0000	0.000
188	CL188	23250	155	0.022	29.20	Not available	Not available			organelle/mitochondria (173 hits 112%)	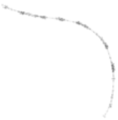	0.0000	0.000
189	CL189	23100	154	0.022	29.20	Not available	Not available	LTR.Copia (**19hits, 9.65%**)	Ty1-RT Ty1/copia AleII (12 hits 7.79%) Ty3-GAG Ty3/gypsy Ogre/Tat (11 hits 7.14%) Ty1-RT Ty1/copia Ivana/Oryco (4 hits 2.6%) Ty1-RT Ty1/copia TAR (2 hits 1.3%) Ty1-RT Ty1/copia AleI/Retrofit (1 hits 0.649%)	organelle/mitochondria (57 hits 37%)	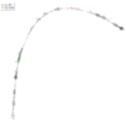	0.0000	0.000
190	CL190	22500	150	0.021	29.30	Not available	Not available	LTR.Gypsy (**79hits, 48.5%**)	Ty3-RH Ty3/gypsy Ogre/Tat (24 hits 16%) Ty3-INT Ty3/gypsy Ogre/Tat (14 hits 9.33%) Ty3-RH Ty3/gypsy Athila (5 hits 3.33%) Ty3-RH Ty3/gypsy chromovirus (3 hits 2%)	organelle/mitochondria (44 hits 29.3%)	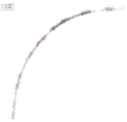	0.0000	0.000
191	CL191	22200	148	0.021	29.30	Not available	Not available			organelle/mitochondria (67 hits 45.3%)	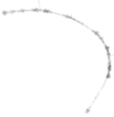	0.0000	0.000
192	CL192	21900	146	0.021	29.30	Not available	Not available			organelle/mitochondria (90 hits 61.6%)	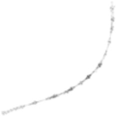	0.0000	0.000
193	CL193	21900	146	0.021	29.30	Not available	Not available			organelle/mitochondria (58 hits 39.7%)	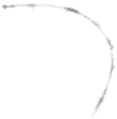	0.0000	0.000
194	CL194	21900	146	0.021	29.30	Not available	Not available	Simple_repeat (9hits, 2.15%) LTR.Copia (1hits, 0.247%) Low_complexity (1hits, 0.242%)			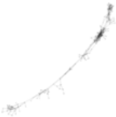	0.1821	0.685
195	CL195	21600	144	0.021	29.40	Not available	Not available	LTR.Gypsy (**142hits, 92.9%**)	Ty3-INT Ty3/gypsy Athila (74 hits 51.4%) Ty3-RH Ty3/gypsy Athila (27 hits 18.8%)		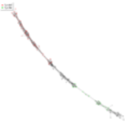	0.0000	0.000

196	CL196	21600	144	0.021	29.40	Not available	Not available			organelle/mitochondria (154 hits 107%)	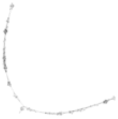	0.1873	0.694
197	CL197	21450	143	0.020	29.40	Not available	Not available			organelle/mitochondria (55 hits 38.5%)	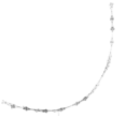	0.0000	0.000
198	CL198	20700	138	0.020	29.40	Not available	Not available	Simple_repeat (10hits, 1.06%)		organelle/mitochondria (138 hits 100%)	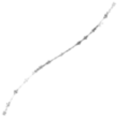	0.0000	0.000
199	CL199	20550	137	0.020	29.40	Not available	Not available	Simple_repeat (2hits, 0.569%)			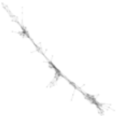	0.5093	2.190
200	CL200	20400	136	0.019	29.50	Not available	Not available	LINE.L1 (**11hits, 4.24%**)	LINE-ENDO NA NA (26 hits 19.1%)	organelle/mitochondria (81 hits 59.6%)	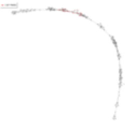	0.0000	0.000
201	CL201	20250	135	0.019	29.50	Not available	Not available			organelle/mitochondria (93 hits 68.9%)	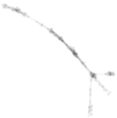	0.0000	0.000
202	CL202	20100	134	0.019	29.50	Not available	Not available	LTR.Gypsy (**44hits, 26.2%**) Simple_repeat (7hits, 1.18%)	Ty3-RT Ty3/gypsy Ogre/Tat (28 hits 20.9%) Ty3-PROT Ty3/gypsy Ogre/Tat (10 hits 7.46%)	organelle/mitochondria (10 hits 7.46%)	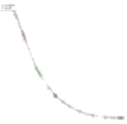	0.0000	0.000
203	CL203	19950	133	0.019	29.50	Not available	Not available	LINE.L1 (**36hits, 21%**) Simple_repeat (2hits, 0.401%) Low_complexity (1hits, 0.165%)			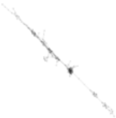	0.0000	0.000
204	CL204	19950	133	0.019	29.50	Not available	Not available			organelle/mitochondria (50 hits 37.6%)	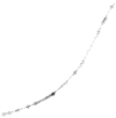	0.0000	0.000
205	CL205	19800	132	0.019	29.60	Not available	Not available			organelle/mitochondria (70 hits 53%)	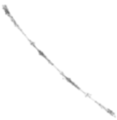	0.0000	0.000
206	CL206	19800	132	0.019	29.60	Not available	Not available	LTR.Copia (**49hits, 30.8%**) Simple_repeat (16hits, 2.95%)	DHH-CD1 NA NA (1 hits 0.758%)		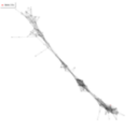	0.1269	0.758

207	CL207	19800	132	0.019	29.60	Not available	Not available	LTR.Copia (**12hits, 4.28%**)		organelle/mitochondria (82 hits 62.1%)	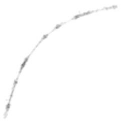	0.0000	0.000
208	CL208	19800	132	0.019	29.60	Not available	Not available			organelle/mitochondria (105 hits 79.5%)	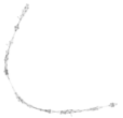	0.0000	0.000
209	CL209	19800	132	0.019	29.60	Not available	Not available	Simple_repeat (14hits, 1.53%)		organelle/mitochondria (94 hits 71.2%)	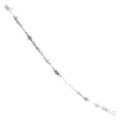	0.0000	0.000
210	CL210	19800	132	0.019	29.70	Not available	Not available	LTR.Copia (**78hits, 44.5%**) Low_complexity (4hits, 1.31%) Simple_repeat (4hits, 1.15%)	Ty1-GAG Ty1/copia Angela (51 hits 38.6%)		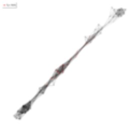	2.2040	9.848
211	CL211	19650	131	0.019	29.70	Not available	Not available	LTR.Gypsy (1hits, 0.193%)		organelle/mitochondria (83 hits 63.4%)	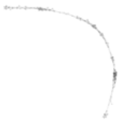	0.0000	0.000
212	CL212	19500	130	0.019	29.70	Not available	Not available			organelle/mitochondria (135 hits 104%)	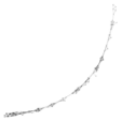	0.0000	0.000
213	CL213	19500	130	0.019	29.70	Not available	Not available	LTR.Copia (**18hits, 11.5%**) Simple_repeat (7hits, 1.46%)	Ty1-GAG Ty1/copia Angela (8 hits 6.15%)	organelle/mitochondria (107 hits 82.3%)		0.0000	0.000
214	CL214	19500	130	0.019	29.70	Not available	Not available			organelle/mitochondria (95 hits 73.1%) organelle/plastid (6 hits 4.62%)	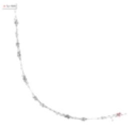 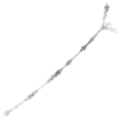	0.3030	1.538
215	CL215	19200	128	0.018	29.70	Not available	Not available	LTR.Copia (**58hits, 33.7%**)	Ty1-INT Ty1/copia AleII (30 hits 23.4%) Ty1-PROT Ty1/copia AleII (8 hits 6.25%) Ty1-PROT Ty1/copia Ivana/Oryco (4 hits 3.12%) Ty1-GAG Ty1/copia AleII (2 hits 1.56%) Ty1-INT Ty1/copia AleI/Retrofit (2 hits 1.56%)		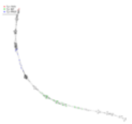	0.0000	0.000
216	CL216	19200	128	0.018	29.80	Not available	Not available	Simple_repeat (9hits, 1.81%)		organelle/mitochondria (127 hits 99.2%)	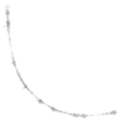	0.0000	0.000

217	CL217	19050	127	0.018	29.80	Not available	Not available	LTR.Copia (**70hits, 46%**)	Ty1-RH Ty1/copia AleII (22 hits 17.3%) Ty1-RT Ty1/copia AleII (10 hits 7.87%) Ty1-RT Ty1/copia unclass(Ale) (6 hits 4.72%) Ty1-RH Ty1/copia AleI/Retrofit (5 hits 3.94%) Ty1-RT Ty1/copia Ivana/Oryco (1 hits 0.787%)	organelle/mitochondria (5 hits 3.94%)	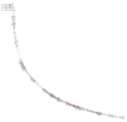	0.0000	0.000
218	CL218	19050	127	0.018	29.80	Not available	Not available	LTR.Gypsy (**88hits, 61.1%**)	Ty3-PROT Ty3/gypsy Athila (38 hits 29.9%) Ty3-PROT Ty3/gypsy Ogre/Tat (1 hits 0.787%)		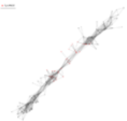	0.0000	0.000
219	CL219	18900	126	0.018	29.80	Not available	Not available	Simple_repeat (10hits, 0.857%)		organelle/mitochondria (92 hits 73%)	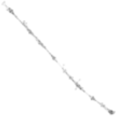	0.0000	0.000
220	CL220	18900	126	0.018	29.80	Not available	Not available	LTR.Copia (**10hits, 3.12%**)	Ty1-INT Ty1/copia AleII (7 hits 5.56%) Ty1-INT Ty1/copia AleI/Retrofit (1 hits 0.794%)	organelle/mitochondria (91 hits 72.2%)	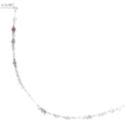	0.0000	0.000
221	CL221	18750	125	0.018	29.90	Not available	Not available	Simple_repeat (2hits, 0.272%)			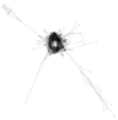	0.0778	0.800
222	CL222	18750	125	0.018	29.90	Not available	Not available			organelle/mitochondria (126 hits 101%)	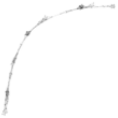	0.0000	0.000
223	CL223	18750	125	0.018	29.90	Not available	Not available			organelle/mitochondria (99 hits 79.2%)	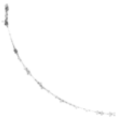	0.0000	0.000
224	CL224	18750	125	0.018	29.90	Not available	Not available	LTR.Copia (**100hits, 75.6%**)	Ty1-RT Ty1/copia AleII (32 hits 25.6%) Ty1-RH Ty1/copia AleII (13 hits 10.4%) Ty1-RH Ty1/copia AleI/Retrofit (10 hits 8%) Ty1-RT Ty1/copia unclass(Ale) (10 hits 8%) Ty1-RH Ty1/copia unclass(Ale) (1 hits 0.8%)......	organelle/mitochondria (8 hits 6.4%)	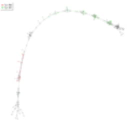	0.0000	0.000
225	CL225	18450	123	0.018	29.90	Not available	Not available	Simple_repeat (2hits, 0.672%) Low_complexity (1hits, 0.255%)			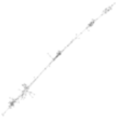	0.0000	0.000
							[illegible]			[illegible]	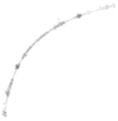		

226	CL226	18450	123	0.018	29.90	Not available	Not available			organelle/mitochondria (49 hits 39.8%)		0.0000	0.000
227	CL227	18450	123	0.018	30.00	Not available	Not available			organelle/mitochondria (123 hits 100%)	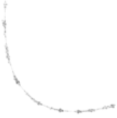	0.0000	0.000
228	CL228	18300	122	0.017	30.00	Not available	Not available	LTR.Gypsy (**46hits, 27.9%**) Simple_repeat (2hits, 0.623%) Low_complexity (1hits, 0.251%)			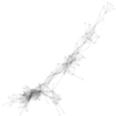	0.7561	2.459
229	CL229	18000	120	0.017	30.00	Not available	Not available	LTR.Copia (**104hits, 81.2%**) Low_complexity (1hits, 0.2%)	Ty1-INT Ty1/copia Angela (39 hits 32.5%) Ty1-RT Ty1/copia Angela (19 hits 15.8%) Ty1-INT Ty1/copia TAR (2 hits 1.67%) Ty1-PROT Ty1/copia Angela (2 hits 1.67%) Ty1-INT Ty1/copia AleII (1 hits 0.833%) Ty1-PR......		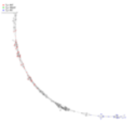	0.2208	0.833
230	CL230	17700	118	0.017	30.00	Not available	Not available	Simple_repeat (2hits, 0.633%)			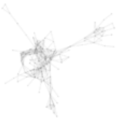	0.0000	0.000
231	CL231	17550	117	0.017	30.00	Not available	Not available	Simple_repeat (13hits, 2.74%) LTR.Copia (3hits, 0.786%)		organelle/mitochondria (93 hits 79.5%)	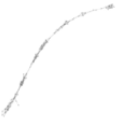	0.0000	0.000
232	CL232	17550	117	0.017	30.00	Not available	Not available	LINE.L1 (**20hits, 12.5%**)			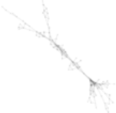	0.0000	0.000
233	CL233	17550	117	0.017	30.10	Not available	Not available			organelle/mitochondria (20 hits 17.1%)	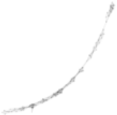	0.0000	0.000
234	CL234	17550	117	0.017	30.10	Not available	Not available	Simple_repeat (**20hits, 5.36%**) Low_complexity (5hits, 2.13%)			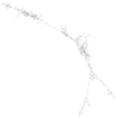	0.0000	0.000
235	CL235	17400	116	0.017	30.10	Not available	Not available	LTR.Gypsy (**6hits, 4.28%**)	Ty3-INT Ty3/gypsy Ogre/Tat (14 hits 12.1%)	organelle/mitochondria (70 hits 60.3%)	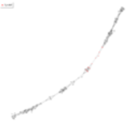	0.0000	0.000
236	CL236	17400	116	0.017	30.10	Not available	Not available	Simple_repeat (7hits, 1.06%) LTR.Copia (1hits, 0.397%) Low_complexity				0.2635	1.724

								(1hits, 0.264%)			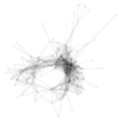		
237	CL237	17250	115	0.016	30.10	Not available	Not available	LINE.L1 (**17hits, 10.8%**)	DHH-CD2 NA NA (1 hits 0.87%)		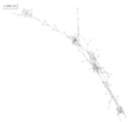	0.0000	0.000
238	CL238	17250	115	0.016	30.10	Not available	Not available			organelle/mitochondria (78 hits 67.8%)	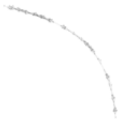	0.0000	0.000
239	CL239	17100	114	0.016	30.20	Not available	Not available	LTR.Copia (**16hits, 6.67%**)<br>LINE.L1 (**12hits, 4.56%**)<br>Simple_repeat (6hits, 1.29%)<br>Low_complexity (4hits, 0.871%)<br>DNA.MULE.MuDR (1hits, 0.655%)<br>LTR.Gypsy (1hits, 0.175%)			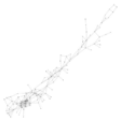	0.0000	0.000
240	CL240	17100	114	0.016	30.20	Not available	Not available			organelle/mitochondria (61 hits 53.5%)	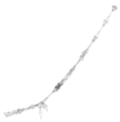	0.0000	0.000
241	CL241	16950	113	0.016	30.20	Not available	Not available			organelle/mitochondria (25 hits 22.1%)	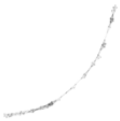	0.0000	0.000
242	CL242	16800	112	0.016	30.20	Not available	Not available			organelle/mitochondria (127 hits 113%)	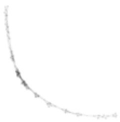	0.0000	0.000
243	CL243	16650	111	0.016	30.20	Not available	Not available	LTR.Caulimovirus (**99hits, 82.2%**)<br>LTR.Gypsy (1hits, 0.775%)	PARA-RT NA NA (28 hits 25.2%)<br>PARA-RH NA NA (8 hits 7.21%)<br>Ty3-RH Ty3/gypsy Athila (6 hits 5.41%)<br>Ty3-RT Ty3/gypsy chromovirus (6 hits 5.41%)<br>Ty3-RH Ty3/gypsy chromovirus (2 hits 1.8%)<br>Ty1-RH Ty1/copia Iva......	organelle/mitochondria (3 hits 2.7%)	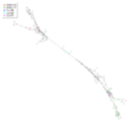	0.0000	0.000
244	CL244	16650	111	0.016	30.20	Not available	Not available	LTR.Copia (**111hits, 95.2%**)	Ty1-PROT Ty1/copia Angela (17 hits 15.3%)<br>Ty1-INT Ty1/copia Angela (7 hits 6.31%)<br>Ty1-RT Ty1/copia AleII (3 hits 2.7%)<br>Ty1-RT Ty1/copia Angela (2 hits 1.8%)		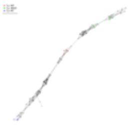	0.3937	0.901
								Simple_repeat (**12hits**					

245	CL245	16650	111	0.016	30.30	Not available	Not available	Simple_repeat (**12hits, 3.6%**) Low_complexity (6hits, 2.34%) LTR.Gypsy (1hits, 0.517%)			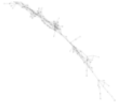	0.0000	0.000
246	CL246	16500	110	0.016	30.30	Not available	Not available			organelle/mitochondria (105 hits 95.5%)	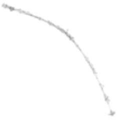	0.0000	0.000
247	CL247	16500	110	0.016	30.30	Not available	Not available	LTR.Gypsy (**110hits, 96.6%**)	Ty3-RH Ty3/gypsy chromovirus (55 hits 50%) Ty3-RT Ty3/gypsy chromovirus (29 hits 26.4%)	organelle/mitochondria (1 hits 0.909%)	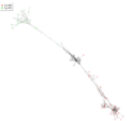	0.0000	0.000
248	CL248	16350	109	0.016	30.30	Not available	Not available	LTR.Copia (**109hits, 97.4%**)	Ty1-INT Ty1/copia Tork (50 hits 45.9%) Ty1-RT Ty1/copia Tork (12 hits 11%) Ty1-PROT Ty1/copia Tork (4 hits 3.67%)		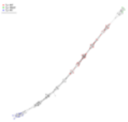	0.0000	0.000
249	CL249	15900	106	0.015	30.30	Not available	Not available			organelle/mitochondria (80 hits 75.5%)	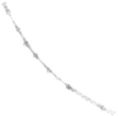	0.4577	1.887
250	CL250	15900	106	0.015	30.30	Not available	Not available	Simple_repeat (6hits, 1.35%) Low_complexity (4hits, 0.95%)		organelle/mitochondria (44 hits 41.5%)	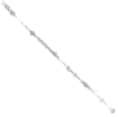	0.0000	0.000
251	CL251	15900	106	0.015	30.40	Not available	Not available	LTR.Copia (**68hits, 55.9%**)	Ty1-RH Ty1/copia Angela (27 hits 25.5%) Ty1-RH Ty1/copia AleI/Retrofit (3 hits 2.83%) Ty1-RT Ty1/copia Angela (3 hits 2.83%) Ty1-RH Ty1/copia Tork (1 hits 0.943%)		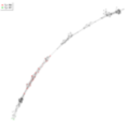	0.2299	0.943
252	CL252	15900	106	0.015	30.40	Not available	Not available	Simple_repeat (**25hits, 11.3%**) LTR.Gypsy (1hits, 0.83%)				0.0000	0.000
253	CL253	15900	106	0.015	30.40	Not available	Not available			organelle/mitochondria (13 hits 12.3%)	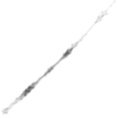	0.0000	0.000
254	CL254	15900	106	0.015	30.40	Not available	Not available	LTR.Gypsy (**30hits, 23.7%**)	Ty3-RH Ty3/gypsy chromovirus (9 hits 8.49%) Ty3-RT Ty3/gypsy chromovirus (8 hits 7.55%)	organelle/mitochondria (43 hits 40.6%)	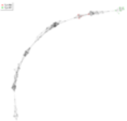	0.0000	0.000
255	CL255	15900	106	0.015	30.40	Not available	Not available			organelle/mitochondria (129 hits 122%)	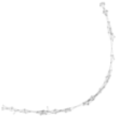	0.0000	0.000
256	CL256	15750	105	0.015	30.40	Not available	Not available	Simple_repeat (10hits, 1.14%) LINE.L1 (1hits, 0.692%)	LINE-ENDO NA NA (12 hits 11.4%)	organelle/mitochondria (104 hits 99%)	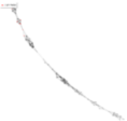	0.0000	0.000

257	CL257	15750	105	0.015	30.40	Not available	Not available	Simple_repeat (5hits, 0.73%)	DTM-CD1 NA NA (1 hits 0.952%)	organelle/mitochondria (75 hits 71.4%)	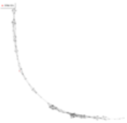	0.0000	0.000
258	CL258	15600	104	0.015	30.50	Not available	Not available	LTR.Gypsy (**28hits, 19.7%**)	Ty3-INT Ty3/gypsy chromovirus (24 hits 23.1%)<br>Ty3-RH Ty3/gypsy chromovirus (6 hits 5.77%)	organelle/plastid (10 hits 9.62%)	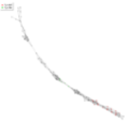	0.0000	0.000
259	CL259	15600	104	0.015	30.50	Not available	Not available	LTR.Gypsy (**12hits, 6.63%**)<br>Simple_repeat (1hits, 0.301%)			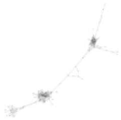	0.0000	0.000
260	CL260	15450	103	0.015	30.50	Not available	Not available	LTR.Gypsy (3hits, 1.26%)<br>Simple_repeat (4hits, 1.1%)<br>Low_complexity (3hits, 0.796%)	Ty3-PROT Ty3/gypsy Ogre/Tat (6 hits 5.83%)	organelle/mitochondria (90 hits 87.4%)	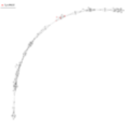	0.0000	0.000
261	CL261	15450	103	0.015	30.50	Not available	Not available	LTR.Gypsy (**21hits, 19.7%**)	Ty3-RT Ty3/gypsy chromovirus (21 hits 20.4%)		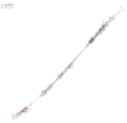	0.0000	0.000
262	CL262	15300	102	0.015	30.50	Not available	Not available	LINE.L1 (**17hits, 9.8%**)<br>Low_complexity (1hits, 0.307%)<br>Simple_repeat (1hits, 0.17%)			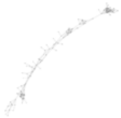	0.0000	0.000
263	CL263	15150	101	0.014	30.50	Not available	Not available	DNA.MULE.MuDR (4hits, 1.67%)<br>DNA.CMC.EnSpm (3hits, 1.15%)<br>Simple_repeat (1hits, 0.152%)			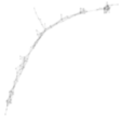	0.0000	0.000
264	CL264	15150	101	0.014	30.50	Not available	Not available	Simple_repeat (5hits, 0.858%)		organelle/mitochondria (84 hits 83.2%)	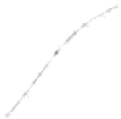	0.0000	0.000
265	CL265	15000	100	0.014	30.60	Not available	Not available	LTR.Copia (**54hits, 32.4%**)	Ty1-INT Ty1/copia AleII (34 hits 34%)<br>Ty1-PROT Ty1/copia AleII (9 hits 9%)<br>Ty1-INT Ty1/copia AleI/Retrofit (2 hits 2%)		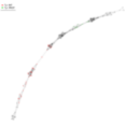	0.0000	0.000
266	CL266	15000	100	0.014	30.60	Not available	Not available	LTR.Copia (8hits, 2.81%)<br>Simple_repeat (10hits, 2.66%)<br>Low_complexity (4hits, 1.04%)	Ty1-PROT Ty1/copia Tork (7 hits 7%)	organelle/mitochondria (52 hits 52%)	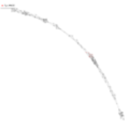	0.0000	0.000
267	CL267	14850	99	0.014	30.60	Not available	Not available				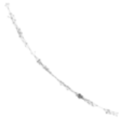	0.0000	0.000

268	CL268	14700	98	0.014	30.60	Not available	Not available	Simple_repeat (2hits, 0.367%)			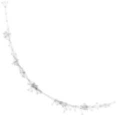	0.0000	0.000
269	CL269	14250	95	0.014	30.60	Not available	Not available			organelle/mitochondria (44 hits 46.3%)	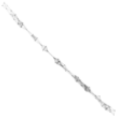	0.0000	0.000
270	CL270	14250	95	0.014	30.60	Not available	Not available			organelle/mitochondria (104 hits 109%)	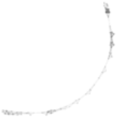	0.0000	0.000
271	CL271	14100	94	0.013	30.60	Not available	Not available			organelle/mitochondria (47 hits 50%) organelle/plastid (2 hits 2.13%)	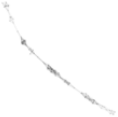	0.0000	0.000
272	CL272	14100	94	0.013	30.70	Not available	Not available	LTR.Copia (**78hits, 76.6%**)	Ty1-RT Ty1/copia Tork (47 hits 50%) Ty1-RT Ty1/copia Ivana/Oryco (4 hits 4.26%)	organelle/mitochondria (4 hits 4.26%)	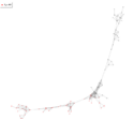	0.0000	0.000
273	CL273	14100	94	0.013	30.70	Not available	Not available	Simple_repeat (1hits, 0.22%)		organelle/mitochondria (50 hits 53.2%)	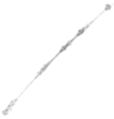	0.0000	0.000
274	CL274	13950	93	0.013	30.70	Not available	Not available			organelle/mitochondria (92 hits 98.9%)	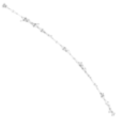	0.0000	0.000
275	CL275	13950	93	0.013	30.70	Not available	Not available			organelle/mitochondria (87 hits 93.5%)	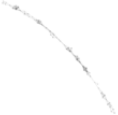	0.0000	0.000
276	CL276	13800	92	0.013	30.70	Not available	Not available	LTR.Copia (**71hits, 56%**)	Ty1-GAG Ty1/copia Ivana/Oryco (35 hits 38%)		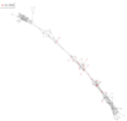	0.0000	0.000
277	CL277	13650	91	0.013	30.70	Not available	Not available			organelle/mitochondria (75 hits 82.4%) organelle/plastid (3 hits 3.3%)	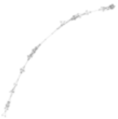	0.0000	0.000
278	CL278	13500	90	0.013	30.70	Not available	Not available	LTR.Copia (**24hits, 17.7%**)	Ty1-PROT Ty1/copia Angela (6 hits 6.67%)		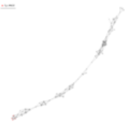	0.0000	0.000

279	CL279	13350	89	0.013	30.70	Not available	Not available			organelle/mitochondria (113 hits 127%)	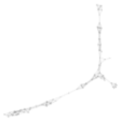	0.3584	1.124
280	CL280	13200	88	0.013	30.80	Not available	Not available			organelle/mitochondria (42 hits 47.7%)	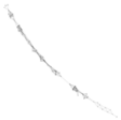	0.0000	0.000
281	CL281	13200	88	0.013	30.80	Not available	Not available				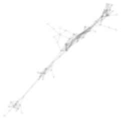	0.0000	0.000
282	CL282	13200	88	0.013	30.80	Not available	Not available			organelle/mitochondria (31 hits 35.2%)	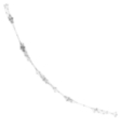	0.0000	0.000
283	CL283	13050	87	0.012	30.80	Not available	Not available	LTR.Gypsy (**47hits, 44.7%**)	Ty3-GAG Ty3/gypsy chromovirus (24 hits 27.6%)		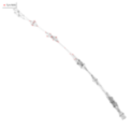	0.0000	0.000
284	CL284	13050	87	0.012	30.80	Not available	Not available	Simple_repeat (4hits, 1.1%) Low_complexity (4hits, 1.1%)		organelle/mitochondria (64 hits 73.6%)	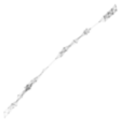	0.0000	0.000
285	CL285	13050	87	0.012	30.80	Not available	Not available			organelle/mitochondria (98 hits 113%)	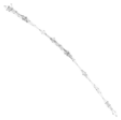	0.0000	0.000
286	CL286	12900	86	0.012	30.80	Not available	Not available	LTR.Copia (**68hits, 76%**)	Ty1-RH Ty1/copia Angela (31 hits 36%) Ty1-RT Ty1/copia Angela (5 hits 5.81%) Ty1-RT Ty1/copia Tork (4 hits 4.65%) Ty1-RH Ty1/copia AleI/Retrofit (3 hits 3.49%) Ty1-RH Ty1/copia Tork (2 hits 2.33%)		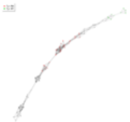	0.0000	0.000
287	CL287	12900	86	0.012	30.80	Not available	Not available	LTR (**45hits, 20.9%**) LTR.Cassandra (**19hits, 9.68%**) LTR.Gypsy (1hits, 0.225%)			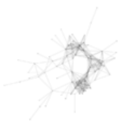	0.0000	0.000
288	CL288	12750	85	0.012	30.90	Not available	Not available			organelle/mitochondria (35 hits 41.2%)	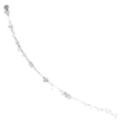	0.0000	0.000

289	CL289	12600	84	0.012	30.90	Not available	Not available	LINE.L1 (**28hits, 23.9%**)	LINE-RT NA NA (45 hits 53.6%)	organelle/mitochondria (84 hits 100%)	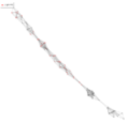	0.9615	2.381
290	CL290	12600	84	0.012	30.90	Not available	Not available	LTR.Copia (**7hits, 6.24%**) Simple_repeat (5hits, 2.13%)			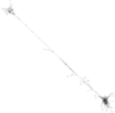	0.0000	0.000
291	CL291	12450	83	0.012	30.90	Not available	Not available	Simple_repeat (2hits, 0.578%)				2.3670	3.614
292	CL292	12450	83	0.012	30.90	Not available	Not available			organelle/mitochondria (4 hits 4.82%)	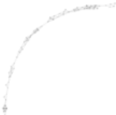	0.0000	0.000
293	CL293	12450	83	0.012	30.90	Not available	Not available	Low_complexity (7hits, 2.28%) Simple_repeat (5hits, 1.55%) Satellite (6hits, 1.49%)			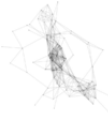	0.0000	0.000
294	CL294	12450	83	0.012	30.90	Not available	Not available	Simple_repeat (5hits, 2.24%) Low_complexity (5hits, 1.86%)			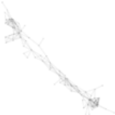	0.0000	0.000
295	CL295	12450	83	0.012	30.90	Not available	Not available	Simple_repeat (4hits, 0.827%) Low_complexity (1hits, 0.281%)			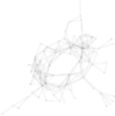	0.0000	0.000
296	CL296	12300	82	0.012	31.00	Not available	Not available			organelle/mitochondria (96 hits 117%)	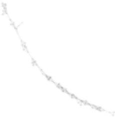	0.0000	0.000
297	CL297	12300	82	0.012	31.00	Not available	Not available	LTR.Copia (**89hits, 72.3%**)	Ty1-INT Ty1/copia AleII (42 hits 51.2%) Ty1-PROT Ty1/copia AleII (5 hits 6.1%)		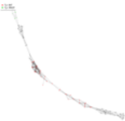	0.0000	0.000
298	CL298	12150	81	0.012	31.00	Not available	Not available	Simple_repeat (1hits, 0.23%)			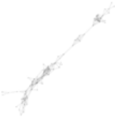	0.0000	0.000
299	CL299	12000	80	0.011	31.00	Not available	Not available	LTR.Copia (**74hits, 86.9%**) Simple_repeat (5hits, 0.833%)	Ty1-INT Ty1/copia Ivana/Oryco (41 hits 51.2%) Ty1-INT Ty1/copia AleII (5 hits 6.25%) Ty1-INT Ty1/copia TAR (1 hits 1.25%) Ty1-INT Ty1/copia Tork (1 hits 1.25%)		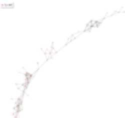	0.0000	0.000

300	CL300	12000	80	0.011	31.00	Not available	Not available			organelle/mitochondria (28 hits 35%)	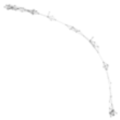	0.0000	0.000
301	CL301	12000	80	0.011	31.00	Not available	Not available			organelle/mitochondria (16 hits 20%)	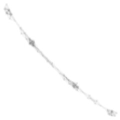	0.0000	0.000
302	CL302	11850	79	0.011	31.00	Not available	Not available			organelle/mitochondria (46 hits 58.2%)	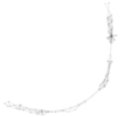	0.0000	0.000
303	CL303	11700	78	0.011	31.00	Not available	Not available	Low_complexity (3hits, 0.923%) Simple_repeat (1hits, 0.308%)		organelle/mitochondria (57 hits 73.1%)	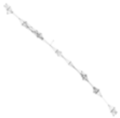	0.0000	0.000
304	CL304	11700	78	0.011	31.00	Not available	Not available					0.0000	0.000
305	CL305	11700	78	0.011	31.10	Not available	Not available			organelle/mitochondria (94 hits 121%)	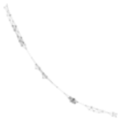	0.0000	0.000
306	CL306	11550	77	0.011	31.10	Not available	Not available			organelle/mitochondria (71 hits 92.2%)	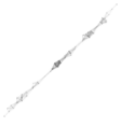	0.0000	0.000
307	CL307	11550	77	0.011	31.10	Not available	Not available	Low_complexity (3hits, 0.987%) Simple_repeat (1hits, 0.303%)		organelle/mitochondria (27 hits 35.1%)	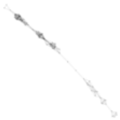	0.0000	0.000
308	CL308	11550	77	0.011	31.10	Not available	Not available	LTR.Copia (**61hits, 68.1%**)	Ty1-INT Ty1/copia unclass(Ale) (18 hits 23.4%) Ty1-INT Ty1/copia AleII (13 hits 16.9%) Ty1-PROT Ty1/copia AleI/Retrofit (5 hits 6.49%) Ty1-INT Ty1/copia AleI/Retrofit (4 hits 5.19%) Ty1-INT Ty1/copia Angela (......		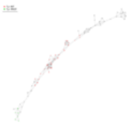	0.0000	0.000
309	CL309	11550	77	0.011	31.10	Not available	Not available	LTR.Gypsy (4hits, 2.35%)	Ty3-RH Ty3/gypsy chromovirus (1 hits 1.3%)	organelle/mitochondria (96 hits 125%)	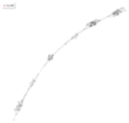	0.0000	0.000
											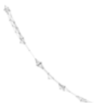		

310	CL310	11400	76	0.011	31.10	Not available	Not available			organelle/mitochondria (63 hits 82.9%)		0.0000	0.000
311	CL311	11400	76	0.011	31.10	Not available	Not available	LTR.Copia (**72hits, 86.1%**)	Ty1-RH Ty1/copia Tork (27 hits 35.5%)<br>Ty1-RT Ty1/copia Tork (23 hits 30.3%)<br>Ty1-RH Ty1/copia Ivana/Oryco (2 hits 2.63%)		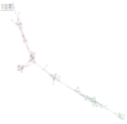	0.0000	0.000
312	CL312	11250	75	0.011	31.10	Not available	Not available	LTR.Copia (**74hits, 94.6%**)	Ty1-RH Ty1/copia Tork (43 hits 57.3%)<br>Ty1-RT Ty1/copia Tork (8 hits 10.7%)	organelle/mitochondria (9 hits 12%)	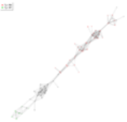	0.0000	0.000
313	CL313	11250	75	0.011	31.10	Not available	Not available	DNA.hAT.Ac (**34hits, 35.7%**)<br>Simple_repeat (2hits, 0.587%)	DTA-CD1 NA NA (23 hits 30.7%)		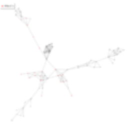	0.0000	0.000
314	CL314	11100	74	0.011	31.20	Not available	Not available			organelle/mitochondria (74 hits 100%)	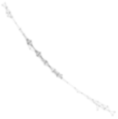	0.0000	0.000
315	CL315	11100	74	0.011	31.20	Not available	Not available	LINE.L1 (**29hits, 24.1%**)<br>Simple_repeat (1hits, 0.216%)			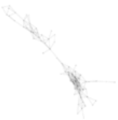	0.0000	0.000
316	CL316	10950	73	0.010	31.20	Not available	Not available	LTR.Gypsy (**42hits, 54.9%**)	Ty3-RT Ty3/gypsy Ogre/Tat (20 hits 27.4%)<br>Ty3-RH Ty3/gypsy Athila (1 hits 1.37%)	organelle/mitochondria (8 hits 11%)	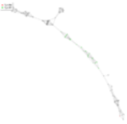	0.0000	0.000
317	CL317	10950	73	0.010	31.20	Not available	Not available	Low_complexity (6hits, 1.59%)		organelle/mitochondria (5 hits 6.85%)	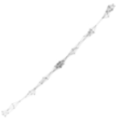	0.0000	0.000
318	CL318	10650	71	0.010	31.20	Not available	Not available			organelle/mitochondria (60 hits 84.5%)	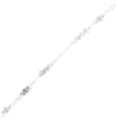	0.0000	0.000
319	CL319	10650	71	0.010	31.20	Not available	Not available			organelle/mitochondria (11 hits 15.5%)	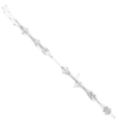	0.0000	0.000
320	CL320	10650	71	0.010	31.20	Not available	Not available	LTR.Copia (**11hits, 6.27%**)		organelle/mitochondria (73 hits 103%)		0.0000	0.000

											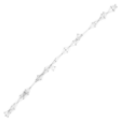		
321	CL321	10650	71	0.010	31.20	Not available	Not available	LTR.Copia (**10hits, 11.2%**)		organelle/mitochondria (8 hits 11.3%)	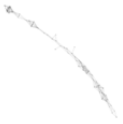	0.0000	0.000
322	CL322	10650	71	0.010	31.20	Not available	Not available	LTR.Caulimovirus (**8hits, 7.73%**) Simple_repeat (**13hits, 6.3%**) LTR.Copia (2hits, 0.77%) Low_complexity (1hits, 0.732%)			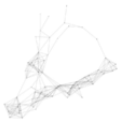	0.0000	0.000
323	CL323	10500	70	0.010	31.20	Not available	Not available	Simple_repeat (1hits, 0.2%)	DTM-CD1 NA NA (40 hits 57.1%) LINE-RT NA NA (1 hits 1.43%)		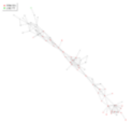	0.0000	0.000
324	CL324	10500	70	0.010	31.30	Not available	Not available	Simple_repeat (8hits, 1.89%)		organelle/mitochondria (1 hits 1.43%)	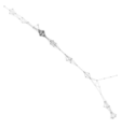	0.0000	0.000
